# Combined, elobixibat, and colestyramine reduced cholesterol toxicity in a mouse model of metabolic dysfunction-associated steatotic liver disease

**DOI:** 10.1097/HC9.0000000000000285

**Published:** 2023-10-31

**Authors:** Michihiro Iwaki, Takaomi Kessoku, Kosuke Tanaka, Anna Ozaki, Yuki Kasai, Takashi Kobayashi, Asako Nogami, Yasushi Honda, Yuji Ogawa, Kento Imajo, Haruki Usuda, Koichiro Wada, Noritoshi Kobayashi, Satoru Saito, Atsushi Nakajima, Masato Yoneda

**Affiliations:** 1 Department of Gastroenterology and Hepatology, Yokohama City University Graduate School of Medicine, Yokohama, Japan; 2 Department of Palliative Medicine, International University Health and Welfare, Narita Hospital, Narita, Japan; 3 Department of Internal Medicine, Asakura Hospital, Konan-ku, Yokohama, Japan; 4 Department of Gastroenterology, National Hospital Organization Yokohama Medical Center, Totsuka-ku, Yokohama, Japan; 5 Department of Gastroenterology, Shinyurigaoka General Hospital, Kawasaki, Japan; 6 Department of Pharmacology, Shimane University Faculty of Medicine, Shimane, Japan; 7 Department of Oncology, Yokohama City University Graduate School of Medicine, Yokohama, Japan

## Abstract

**Background:**

Cholesterol levels and bile acid metabolism are important drivers of metabolic dysfunction-associated steatohepatitis (MASH) progression. Using a mouse model, we investigated the mechanism by which cholesterol exacerbates MASH and the effect of colestyramine (a bile acid adsorption resin) and elobixibat (an apical sodium-dependent bile acid transporter inhibitor) concomitant administration on bile acid adsorption and MASH status.

**Methods:**

Mice were fed a high-fat high-fructose diet with varying concentrations of cholesterol to determine changes in fatty liver according to liver status, water intake, defecation status, insulin resistance, bile acid levels, intestinal permeability, atherosclerosis (in apolipoprotein E knockout mice), and carcinogenesis (in diethylnitrosamine mice). Using small interfering ribonucleic acid (siRNA), we evaluated the effect of sterol regulatory element binding protein 1c (SREBP1c) knockdown on triglyceride synthesis and fatty liver status following the administration of elobixibat (group E), colestyramine (group C), or both (group EC).

**Results:**

We found greater reductions in serum alanine aminotransferase levels, serum lipid parameters, serum primary bile acid concentrations, hepatic lipid levels, and fibrosis area in EC group than in the monotherapy groups. Increased intestinal permeability and watery diarrhea caused by elobixibat were completely ameliorated in group EC. Group EC showed reduced plaque formation rates in the entire aorta and aortic valve of the atherosclerosis model, and reduced tumor counts and tumor burden in the carcinogenesis model.

**Conclusions:**

Excessive free cholesterol in the liver can promote fatty liver disease. Herein, combination therapy with EC effectively reduced free cholesterol levels in MASH model mice. Our study provides strong evidence for combination therapy as an effective treatment for MASH.

## Graphical abstract

**Figure:**
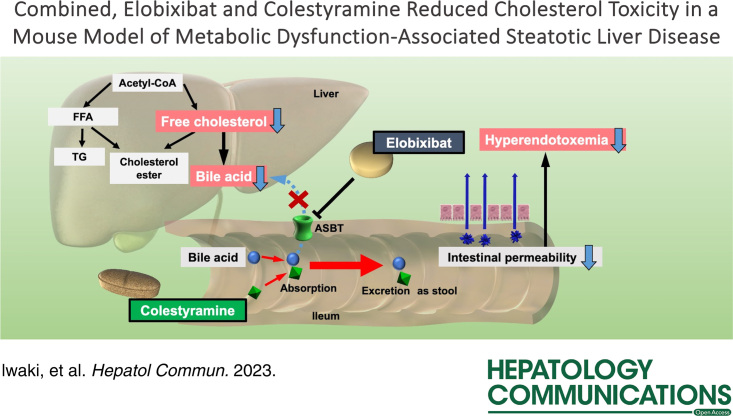

## INTRODUCTION

Metabolic dysfunction-associated steatotic liver disease (MASLD), formerly known as NAFLD, is a hepatic manifestation of metabolic syndrome.^1–3^ MASLD includes metabolic dysfunction-associated steatohepatitis (MASH), which encompasses inflammatory and progressive diseases associated with liver cancer or cirrhosis.^2,4^

Accumulation of triglycerides (TGs) in hepatocytes is a hallmark of MASLD and MASH. While the exact mechanisms are unclear, various factors, including lipotoxicity, inflammation, insulin resistance, and oxidative stress, can promote the progression of steatosis to MASH.^5^ Cholesterol intake is also strongly associated with the progression of MASH.^6^ Since free cholesterol (FC) is highly hepatotoxic, a pathway mediated by TG production maintains constant cholesterol accumulation. Neutral fat accumulation has also been suggested to protect the liver.^7,8^

Effective drug treatments for MASH have not yet been established. Nevertheless, the restoration of normal cholesterol levels is an important part of the treatment strategy. Bile acid synthesis is an important factor in cholesterol excretion. In patients with MASH, the total bile acid concentration in the blood is approximately twice that in healthy individuals.^9^ Impaired bile acid metabolism has recently been shown to contribute to the pathophysiology of metabolic diseases, including MASLD.^10^

Elobixibat—initially developed for hyperlipidemia—is an absorbed inhibitor of the ileal bile acid transporter and the apical sodium-dependent bile acid transporter (ASBT). With the administration of an ASBT inhibitor, bile acids are excreted in stool without being reabsorbed. Therefore, the inhibition of ASBT conceivably reduces bile acid pool size and indirectly stimulates the hepatic production of bile acids from FC.^11^ The main side effect of ASBT inhibitors is diarrhea, also known as bile acid diarrhea, which has promoted their use in Japan for treating chronic constipation. Recent studies using nonabsorbable ASBT inhibitors demonstrated improved liver conditions in rodent models of MASLD.^12^ However, a multicenter phase 2 randomized control trial (NCT02787304) of volixibat was terminated prematurely owing to a lack of efficacy and the occurrence of treatment-emergent adverse events (Philip, N. 2019 EASL Abstract no: 5212). Furthermore, the effects of ASBT inhibitors on MASH have not yet been established.

Colestyramine is an anion-exchange resin or bile acid sequestrant (BAS). BAS absorbs bile acids, decreasing their reabsorption. As the bile acid pool is reduced, cholesterol is converted into bile acid. Therefore, colestyramine has been used to treat hyperlipidemia. The main side effects are constipation and stomach/abdominal fullness. An estimated 70%–96% of patients with chronic diarrhea with bile acid malabsorption respond to short-course cholestyramine.^13^

Colestimide—a BAS—improved steatosis and aspartate aminotransferase levels in 17 patients with MASH with hyperlipidemia receiving 3 g/d.^14^ However, in patients with biopsy-proven MASH, colesevelam at 3.75 g/d for 24 weeks increased liver fat deposition (detected by MRI proton density fat fraction) without affecting steatosis, cellular injury, or lobular inflammation (according to a liver biopsy assessment).^15^ Therefore, the effect of BAS on MASH remains controversial.

Using a mouse model, this study examines the role of cholesterol in MASH progression. We hypothesized that combination therapy with an ASBT inhibitor and BAS might reduce serum bile acid levels and ameliorate diarrhea without increasing portal endotoxin levels, ultimately imparting a beneficial treatment effect.

## METHODS

### Ethics statement

All animals used in this study received humane care according to the standards outlined in the Guide for the Care and Use of Laboratory Animals. Moreover, this study was conducted following the Guidelines for the Care and Use of Laboratory Animals of the Yokohama City University Medical School (Yokohama, Japan). The protocol was approved by the Committee on the Ethics of Animal Experiments of the Yokohama City University Graduate School of Medicine (permit number: F-A-15-016).

### Drugs, animals, and experimental design

#### High-fat high-fructose cholesterol loading model

All animals were housed under conventional conditions with controlled temperature, humidity, and light (12-h light-dark cycle) and provided food and water. After acclimatization with a basal diet (BD), 8-week-old male *C57/B6J* mice (CLEA Japan, Tokyo, Japan) were divided into 5 groups, according to diet, for 20 weeks: a control (BD), high-fat diet (40% kcal, Primex partially hydrogenated vegetable oil shortening), high fructose (22% by weight), and cholesterol (0%, 0.2%, 0.6%, and 2%; Research Diets, New Brunswick, NJ). A mouse loaded with fats (40% kcal), fructose (22% by weight), and 2% cholesterol (2% by weight) is known as an amylin liver MASH (amylin metabolic dysfunction-associated steatohepatitis) model. All surgeries were performed under sodium pentobarbital anesthesia, and all efforts were made to minimize suffering. The experimental protocol is outlined in Supplemental Figure S1A, http://links.lww.com/HC9/A615. Additionally, to examine changes in cholesterol loading over time, 8-week-old male C57/B6J mice (CLEA Japan, Tokyo, Japan) were fed BD, a high-fat diet (40% kcal), a high-fructose diet (22% by weight) with no added cholesterol, and the amylin MASH diet (Research Diets) for 1, 4, and 8 weeks. In each group, liver enzyme levels and lipid content in the liver were determined, and real-time PCR was performed over time. The experimental protocol is outlined in Supplemental Figure S1B, http://links.lww.com/HC9/A615.

#### siRNA knockdown of SREBP1c

Eight-week-old male *C57/B6J* mice (CLEA Japan) were fed an amylin liver MASH diet—enriched in fat (40% kcal, Primex partially hydrogenated vegetable oil shortening), fructose (22% by weight), and cholesterol (2% by weight)—for 20 weeks (Research Diets). For the last 4 weeks, Ambion in vivo siRNA (Thermo Fisher Scientific, Tokyo, Japan) with the antisense of SREBP1c target sequence 5′-UACAUCUUUAAAGCAGCGGGT-3′ were combined with invivofectamine 3.0 (Thermo Fisher Scientific) to form complexes following the manufacturer’s instructions. The complex was injected into MASH model mice by means of the tail vein at 20 µg per mouse every three days for 4 weeks. PBS was used as a control. The experimental protocol is outlined in Supplemental Figure S1C, http://links.lww.com/HC9/A615.

#### Elobixibat and colestyramine treatment in a MASH model

Elobixibat was purchased from EA Pharma (Tokyo, Japan), and colestyramine from Sanofi (Tokyo, Japan). A BD containing 22% protein, 6% fat, and 47% carbohydrate was prepared. An amylin MASH diet—enriched in fat (40% kcal, Primex partially hydrogenated vegetable oil shortening), fructose (22% by weight), and cholesterol (2% by weight)—was purchased from Research Diets. Eight-week-old male *C57BL/6J* mice (CLEA Japan) were divided into groups for 20 weeks according to diet: a BD, amylin MASH, amylin MASH/elobixibat (10 mg/kg/d, week 16–20), amylin MASH/colestyramine (0.5 g/kg/d, week 16–20), and amylin MASH/elobixibat/colestyramine group. The experimental protocol is outlined in Supplemental Figure S1D, http://links.lww.com/HC9/A615.

#### Apolipoprotein E knockout mice

After acclimatization with the BD, 8-week-old male apolipoprotein E knockout [*ApoE*(KO)] mice (CLEA Japan) were divided into groups for 24 weeks according to diet: BD, amylin MASH, amylin MASH/elobixibat (10 mg/kg/d, week 16–24), amylin MASH/colestyramine (0.5 g/kg/d, week 16–24), and amylin MASH/elobixibat/colestyramine groups. The experimental protocol is outlined in Supplemental Figure S2A, http://links.lww.com/HC9/A616. After 24 weeks, the heart and aorta were perfused with saline by means of the left ventricle, removed, and fixed with 4% paraformaldehyde.

#### Diethylnitrosamine model

Male *B6C3F1* mice (CLEA Japan) were intraperitoneally administered 50 mg/kg body weight diethylnitrosamine (Sigma-Aldrich, St. Louis, MO)—an environmental carcinogen that produces mutagenic DNA adducts that cause genetic sequence mismatch and predispose the liver to HCC formation—^16–19^ from 6 to 12 weeks of age, with a 2-week interval between injections. Carcinogenesis was observed after 28 weeks of diethylnitrosamine administration and amylin MASH diet (Supplemental Figure S3, http://links.lww.com/HC9/A617). Liver dissection was performed at 32 weeks of age. The experimental protocol is outlined in Supplemental Figure S2B, http://links.lww.com/HC9/A616.

The groups of mice were fed for 32 weeks as follows: a BD, amylin MASH diet (group MASH), amylin MASH/elobixibat (10 mg/kg/d, week 24–32), amylin MASH/colestyramine (0.5 g/kg/d, week 24–32), or amylin MASH/elobixibat/colestyramine diet. The number of malignant nodules (dysmorphic or dyschromic nodules with a diameter of ≥5 mm) was determined by macroscopic examination of the liver.

### Sample collection and serological examination

The method is described in Appendix 1, http://links.lww.com/HC9/A627.

### Liver histology

The method is described in Appendix 2, http://links.lww.com/HC9/A627.

### Tumor analysis

The diameter of each visible tumor (≥0.5 mm) was used to calculate tumor volume using the formula: 4/3×π*r* ^3^. The sum of individual tumor volumes was considered the tumor burden.

### Evaluation of atherosclerosis

The method is described in Appendix 3, http://links.lww.com/HC9/A627.

### Western blotting

Nuclear proteins were extracted from liver and ileum tissues using a nuclear extraction kit (Abcam, Cambridge, MA) following the manufacturer’s instructions. Proteins were incubated with primary antibodies and horseradish peroxidase–conjugated secondary antibodies (Cell Signaling Technology, Danvers, MA). The primary antibody was farnesoid X receptor (FXR; Cell Signaling Technology; catalog number 72105). Glyceraldehyde 3 phosphate dehydrogenase was used as a loading control for total protein.

### Hepatic and ileal RNA isolation and real-time PCR analyses

The methods are described in Appendix 4, http://links.lww.com/HC9/A627.

### Liquid chromatography-tandem mass spectrometry

The method is described in Appendix 5, http://links.lww.com/HC9/A627

### Statistical analysis

Data are presented as the mean±SE. Differences between 2 groups were assessed using Student *t* test. Statistical significance was set at *p*<0.05. Statistical analyses were performed using JMP software v.14 (SAS Institute, Cary, NC).

## RESULTS

### Effect of cholesterol loading in high-fat high-fructose diet

Table 1 shows the characteristics of mice fed an high-fat high-fructose (HFHF) diet with added cholesterol (0%, 0.2%, 0.6%, and 2%) for 20 weeks. The HFHF diet with no cholesterol increased body weight, liver weight, epididymal weight, and subcutaneous fat compared with the BD. Adding more than 0.2% cholesterol resulted in markedly higher increases in body and liver weights.

**TABLE 1:** Characteristics of mice fed a high-fat high-fructose diet with added cholesterol

Parameters	BD	HFHF+no cholesterol	HFHF+0.2% cholesterol	HFHF+0.6% cholesterol	HFHF+2% cholesterol
Body weight (g)	31.6±0.5	40.6±1.2^a^	45.4±0.7^b^	44.9±0.7^b^	44.6±0.5^b^
Liver weight (g)	1.2±0.03	2.1±0.19^a^	3.7±0.08^b^	3.6±0.14^b^	4.1±0.15^b^
Epididymal adipose tissue weight (g)	0.8±0.08	2.1±0.19^a^	2.2±0.14	2.4±0.14	2.6±0.08^c^
AST (IU/L)	61.3±12.2	138.9±14.7^a^	253.6±48.4	220.9±27.6^c^	277.0±37.0^b^
ALT (IU/L)	22.6±1.8	122.4±11.4^a^	263.6±26.6^b^	264.0±39.4^b^	258.6±37.7^b^
Total cholesterol (mg/dL)	83.7±2.7	204.6±5.9^a^	300.3±10.2^b^	308.3±12.9^b^	270±11.5^b^
Free fatty acid (uEQ/L)	672.1±15.3	727.7±55.7	926.4±59.9^c^	871.8±25.0^c^	1016.9±49.5^b^
Fasting plasma glucose (mg/dL)	111.7±6.1	135.7±8.9^a^	183.6±11.8	174.0±4.5	196.3±15.9^c^
Insulin (ng/mL)	1.7±0.5	5.4±1.1^a^	4.4±0.8	5.3±1.7	7.3±1.8
HOMA-IR	0.5±0.2	1.7±0.3^a^	2.0±0.5	2.3±0.7	4.1±0.9^c^

*Note:* Data are presented as the mean±SE (n=7–8). Significance was determined using Student *t* test.

a *p*<0.05 versus BD group.

b *p*<0.01 versus HFHF + no cholesterol group.

c *p*<0.05 versus HFHF + no cholesterol group.

Abbreviations: ALT, alanine aminotransferase; AST, aspartate aminotransferase; BD, basal diet; HFHF, high-fat high-fructose; HOMA-IR, homeostasis model assessment of insulin resistance.

**FIGURE 1:**
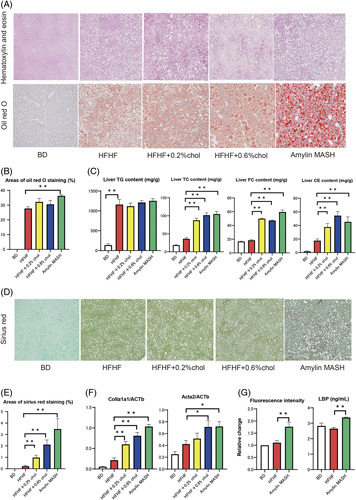
(A) Liver sections from the BD, HFHF, and cholesterol-loaded groups. Representative images of hematoxylin and eosin and Oil Red O staining results. (B) Areas of Oil Red O staining in the livers of mice fed a BD, HFHF, or HFHF diet with added cholesterol (n=6–7). (C) TG, TC, and FC contents were measured in the livers of mice fed a BD, HFHF, or HFHF diet with added cholesterol (n=7–8). (D) Sirius Red staining. (E) Areas of Sirius Red staining in the livers of mice fed a BD, HFHF, or HFHF diet with added cholesterol (n=5–6). (F) Expression of Col1α1 and ACTA2 mRNA in mice fed a BD, HFHF, or HFHF diet supplemented with cholesterol (n=7–8). (G) Fluorescence intensity (n=5) and lipopolysaccharide-binding protein (LBP, n=6–8) levels in mice fed a BD, HFHF, or HFHF diet supplemented with cholesterol. Data are presented as the mean±SE. Significance was determined using Student *t* test. *p<0.05, **p<0.01. Abbreviations: amylin MASH, amylin liver metabolic dysfunction-associated steatohepatitis; BD, basal diet; CE, cholesterol ester; Col1a1, collagen 1α1; FC, free cholesterol; HFHF, high-fat high-fructose; LBP, lipopolysaccharide-binding protein; TC, total cholesterol; TG, triglyceride.

The areas of Oil Red O staining were increased by the HFHF diet, and even more so with the amylin MASH diet (Figure 1A, B). The HFHF diet also increased TG, total cholesterol (TC), and cholesterol ester levels in the liver to about 8-fold, 2-fold, and 9-fold that of the BD diet, respectively (Figure 1C). Adding more than 0.2% cholesterol significantly increased the TC and FC levels in the liver.

Sirius Red staining showed that liver fibrosis increased with cholesterol load. The mRNA expression of collagen 1α1 (Col1α1) and ACTA2—markers of liver fibrosis—also increased with cholesterol load.

Supplemental Figure S4, http://links.lww.com/HC9/A618 shows the changes in plasma bile acid levels with the addition of cholesterol to the HFHF diet. The HFHF diet with 0% or 0.2% cholesterol did not alter the total, primary, or secondary bile acid plasma levels compared with the BD. Compared to the HFHF diet without cholesterol, the amylin MASH diet increased the plasma levels of total, primary, and secondary bile acids by 1.9-fold, 2.1-fold, and 1.4-fold, respectively. Cholic acid (CA), chenodeoxycholic acid (CDCA), and β-muricholic acid plasma levels were highest in the amylin MASH group.

In the cholesterol-loaded groups, total, primary, and secondary bile acids were all elevated in stool samples. CA, deoxycholic acid (DCA), and β-muricholic acid fecal levels were also increased compared to the BD group (Supplemental Figure S5, http://links.lww.com/HC9/A619).

**FIGURE 2:**
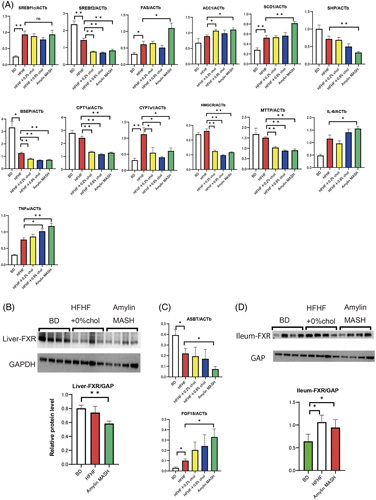
(A) Real-time PCR of the liver samples of mice fed a BD, HFHF, or HFHF with added cholesterol diet (n=5–8). (B) Liver-FXR protein levels in mice fed a BD, HFHF, or HFHF diet supplemented with cholesterol (n=4). (C) Real-time PCR of ileum samples from mice fed a BD, HFHF, or HFHF diet supplemented with cholesterol (n=5–6). (D) Ileum-FXR protein levels in mice fed a BD, HFHF, or HFHF diet supplemented with cholesterol (n=4). Data are presented as the mean±SE. Significance was determined using Student *t* test. *p<0.05, **p<0.01. Abbreviations: ACC1, acetyl-CoA carboxylase 1; amylin MASH, amylin liver metabolic dysfunction-associated steatohepatitis; BD, basal diet; BSEP, bile salt export pump; CYP7a1, cytochrome P450 7A1; FAS, fatty acid synthase; FXR, farnesoid X receptor; HFHF, high-fat high-fructose; MTTP, microsomal triglyceride transfer protein; SCD1, stearoyl-CoA desaturase 1.

Fluorescein isothiocyanate (FITC) and lipopolysaccharide-binding protein (LBP) were used to evaluate intestinal permeability. Figure 1G shows the relative change in fluorescence intensity between diets. Fluorescence intensity was comparable between the HFHF and BD groups but increased significantly in the amylin MASH group. LBP levels were increased by loading more than 0.2% cholesterol compared with the HFHF diet. Furthermore, IL-6 levels were significantly elevated in the amylin MASH group, and TNFα levels were significantly elevated in the 0.6% cholesterol and amylin MASH groups compared with those in the HFHF diet group (Figure 2).

FXR—a nuclear receptor—uses bile acids as ligands to regulate target gene expression. Western blotting of the liver showed that FXR expression was suppressed in the amylin MASH–fed mice (Figure 2B), consistent with the expression of small heterodimer partner and bile salt export pump, which are downstream signals of FXR in the liver. Figure 2C shows the expression of ASBT (suppressed by FXR) and FGF15 (activated by FXR) in ileal PCR. FGF15 mRNA expression was upregulated, whereas ASBT expression was downregulated. Western blotting of the ileum showed that the HFHF and amylin MASH diets upregulated FXR expression compared with BD (Figure 2D).

Regarding the gene expression of proteins involved in lipid synthesis, the HFHF diet increased the mRNA expression of sterol regulatory element-binding transcription factor 1c (SREBf1c) and suppressed that of SREBf2. Cholesterol addition did not alter SREBf1c expression but significantly suppressed SREBf2 expression at concentrations of 0.2% or more. The expression of SREBf1c, fatty acid synthase, acetyl-CoA carboxylase 1, and stearoyl-CoA desaturase 1 was increased by the amylin MASH diet compared with the HFHF diet. The levels of cytochrome P450 7A1 (CYP7a1)—a rate-limiting enzyme involved in hepatic bile acid synthesis from cholesterol—were lower with the BD and cholesterol-loaded diets than with the HFHF diet. Moreover, adding more than 0.2% cholesterol to the HFHF diet significantly reduced the levels of microsomal triglyceride transfer protein, a transporter that excretes cholesterol esters as VLDL from the liver.

Supplemental Figure S6, http://links.lww.com/HC9/A620 shows the time-dependent increase in serum alanine aminotransferase levels and hepatic TG and FC content with continued cholesterol loading. Real-time PCR of the liver showed that SREBP1c, fatty acid synthase, acetyl-CoA carboxylase 1, stearoyl-CoA desaturase 1, and CYP7a1 expression peaked 1 week after cholesterol loading. These changes tend to be alleviated gradually with continued cholesterol loading, whereas SREBP2 expression remains strongly suppressed with continued loading.

### SREBP1c knockdown using siRNA

Using siRNA, we evaluated the effect of SREBP1c knockdown on TG synthesis and fatty liver status. To confirm the efficient knockdown of SREBP1c, we assessed the change in SREBP1c expression 2 days after the first injection of siRNA. We observed a significant knockdown of 52% in the siRNA compared to the control group (fed a normal diet for 16 weeks; Supplemental Figure S1C, http://links.lww.com/HC9/A615).

**FIGURE 3:**
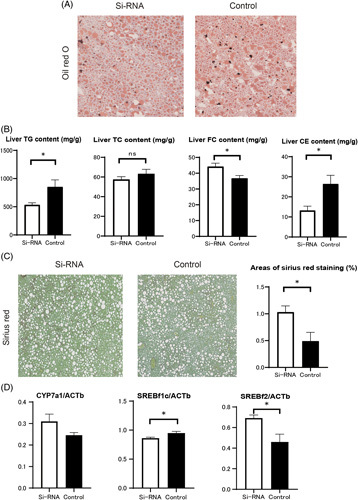
(A) Oil Red O staining of liver sections from SREBP1c knockdown and control mice (n=6–7). (B) TG, TC, and FC contents were measured in the livers of SREBP1c knockdown and control mice (n=6–7). (C) Sirius Red staining and areas of Sirius Red staining in the livers of SREBP1c knockdown and control mice (n=6–7). (D) Real-time PCR of liver samples from SREBP1c knockdown and control mice (n=5). Data are presented as the mean±SE. Significance was determined using Student *t* test. *p<0.05, **p<0.01. Abbreviations: CE, cholesterol ester; CYP7a1, cytochrome P450 7A1; FC, free cholesterol; Si-RNA, small interfering ribonucleic acid; TC, total cholesterol; TG, triglyceride.

Knockdown of SREBP1c resulted in an 8% reduction in body weight (siRNA group, 35.9±0.7; control group, 38.9±0.7, *p*=0.008) and a 16% reduction in liver weight (siRNA group, 3.2±0.2; control group, 3.8±0.2, *p*=0.016; Supplemental Table S1, http://links.lww.com/HC9/A626). There were no statistically significant differences in the weight of the epididymal adipose tissue. SREBP1c knockdown mice had higher levels of TC (siRNA group, 246.0±4.2; control group, 205.5±12.4, *p*=0.021), aspartate aminotransferase (siRNA group, 170.6±15.7; control group, 122.0±14.1, *p*=0.048), and alanine aminotransferase (siRNA group, 244.4±22.1; control group, 157.3±23.3, *p*=0.024). Liver TG and cholesterol ester contents were lower in the siRNA group (Figure 3B). A statistically significant decrease in the levels of blood bile acids, mainly primary bile acids such as CA and CDCA, was observed (Supplemental Figure S7, http://links.lww.com/HC9/A621). In terms of the extent of liver fibrosis, SREBP1c knockdown mice showed marked progression of liver fibrosis (Figure 3C). The expression of Col1α1 and ACTA2 was enhanced by SREBP1c knockdown.

### Efficacy of elobixibat and cholestyramine in the MASH mouse model

Table 2 shows the characteristics of the MASH model mice treated with elobixibat and cholestyramine (EC), for which we observed no significant difference. Water intake was 1.7-fold higher in the elobixibat group than in the cholestyramine group. The number of feces and fecal water content increased 1.8- and 1.6-fold, respectively, in group E compared to group MASH. In the EC group, the number of feces and fecal water content were 44% and 30% lower, respectively, than in group E. Body weight, liver weight, and the weight of epididymal adipose tissue increased 1.5-, 3.2-, and 3.8-fold, respectively, in the MASH group compared with the BD group; compared to the MASH group, they decreased by 10%, 24%, and 13% in group E and by 9%, 12%, and 9% in group C, respectively. The combination of EC reduced these levels by 22%, 52%, and 48%, respectively, compared with the MASH group. Aspartate aminotransferase and alanine aminotransferase levels in the blood were higher in the MASH group, lower in the elobixibat group, and remarkably lower in the EC group compared with the MASH and monotherapy groups. The increase in blood TC and free fatty acid levels in the MASH group was less than half that in the EC group. Insulin resistance, according to a homeostasis model assessment of insulin resistance (HOMA-IR), increased in the MASH group and decreased most remarkably in the EC group.

**TABLE 2:** Characteristics of MASH model treated with elobixibat and cholestyramine

Parameters	Basal diet (group BD)	AMLM (group MASH)	AMLM+Elobixibat (group E)	AMLM+Colestyramine (group C)	AMLM+Elobixibat+colestyramine (group EC)
Food intake (g/d)	3.09±0.09	3.22±0.05	3.38±0.16	3.38±0.11	3.16±0.04
Water intake (mL/d)	5.16±0.28	3.53±0.41^a^	6.23±0.28^b^	4.17±0.15	3.8±0.12
Stool water content (%)	51.75±2.95	36.71±1.72^a^	58.54±1.85^c^	32.63±1.92	40.81±1.12
Stool particle count (particle number/day/mouse)	81.88±4.52	50.38±2.94^a^	89.00±2.71^c^	39.63±2.43^b^	50.25±2.85
Body weight (g)	29.2±0.6	43.3±0.6^a^	38.8±1.3^c^	39.4±0.8^c^	33.8±0.7^c^
Liver weight (g)	1.3±0.03	4.2±0.18^a^	3.2±0.19^c^	3.7±0.11^c^	2±0.10^c^
Epididymal adipose tissue weight (g)	0.6±0.07	2.3±0.11^a^	2.0±0.14	2.1±0.16	1.2±0.12^c^
HOMA-IR	0.41±0.16	2.94±0.71^a^	1.50±0.47	1.42±0.49	0.81±0.45^b^
Total cholesterol (mg/dL)	83.6±2.4	260.4±10.9^a^	224.6±17.3	235.5±15.3	126.1±12.9^c^
AST (IU/L)	57.5±11.2	292.375±37.9^a^	169±14.9^b^	275±63.8	111.3±26.7^b^
ALT (IU/L)	21.8±11.2	239.5±37.9^a^	94.9±14.9^c^	197.3±63.8	32.6±26.7^c^
Free fatty acid (uEQ/L)	723.8±58.6	1061.1±50.5^a^	781.6±60.0^c^	824±55.0^c^	426.8±47.0^c^

*Note:* Data are presented as the mean±SE (n=6–8). Significance was determined using Student *t* test.

a *p*<0.05 versus BD group.

b *p*<0.05 versus MASH group.

c *p*<0.01 versus MASH group.

Abbreviations: ALT, alanine aminotransferase; AMLM, amylin liver metabolic dysfunction-associated steatohepatitis; AST, aspartate aminotransferase; BD, basal diet; C, cholestyramine; E, elobixibat; EC, elobixibat and cholestyramine; HOMA-IR, homeostasis model assessment of insulin resistance; MASH, metabolic dysfunction-associated steatohepatitis.

**FIGURE 4:**
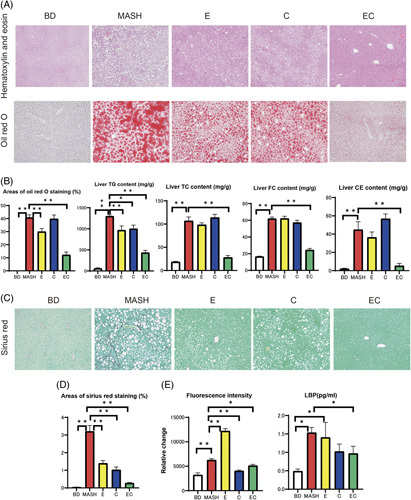
(A) Oil Red O staining results of liver sections from mice fed a basal diet (BD), amylin liver metabolic dysfunction-associated steatohepatitis (amylin MASH) (MASH group), amylin MASH with elobixibat (E) and cholestyramine alone (C), or amylin MASH with a combination of elobixibat and cholestyramine (EC). (B) Oil Red O staining area, triglyceride (TG), total cholesterol (TC), and free cholesterol (FC) contents in the liver samples of mice fed a BD, amylin MASH (MASH group), or amylin MASH with elobixibat and cholestyramine (n=5–7). (C) Sirius Red (SR) staining in the liver. (D) Areas of SR staining in the liver (n=5–7). (E) Fluorescein isothiocyanate and lipopolysaccharide-binding protein (LBP) (n=5–7). Data are presented as the mean±SE. Significance was determined using Student *t* test. * *p*<0.05, ** *p*<0.01. Abbreviations: BD, basal diet; C, cholestyramine; CE, cholesterol ester; E, elobixibat; EC, elobixibat and cholestyramine; FC, free cholesterol; LBP, lipopolysaccharide-binding protein; MASH, metabolic dysfunction-associated steatohepatitis; TC, total cholesterol; TG, triglyceride.

Steatosis and fibrosis of the liver were clearly observed in our histological analysis of the MASH group (Figure 4). The Oil Red O staining area was smaller in group E (*p*=0.006) and group EC (*p*<0.001) mice than in the MASH group. Compared with the MASH group, the area stained by Sirius Red was smaller in groups E (*p*<0.001), C (*p*<0.001), and EC (*p*<0.001). The levels of TC, FC, and TG in liver tissue increased 6-, 4-, and 22-fold, respectively, in the MASH group compared with the BD group. Hepatic TG content decreased in both groups E and C and decreased remarkably in the EC group. For TC and FC, there was no statistical change in group C compared with the MASH group, though there was a statistically significant decrease in group E, and a further decrease in the EC group.

Supplemental Figure S8, http://links.lww.com/HC9/A622 shows the changes in plasma bile acid levels of the MASH model after treatment with EC. The MASH group showed the highest total, primary, and secondary plasma bile acid levels. Elobixibat alone reduced the total and primary bile acid levels by 45% and 61%, respectively. The addition of cholestyramine to the amylin MASH diet did not alter total or primary bile acid levels but reduced secondary bile acid levels by 33%. The combination of EC significantly reduced the total, primary, and secondary bile acid levels compared to the MASH group. As shown in Supplemental Figure S9, http://links.lww.com/HC9/A623, total, primary, and secondary bile acid levels in stool samples were the highest in group E. The combination of cholestyramine and elobixibat reduced the levels of secondary bile acids, but the levels of primary bile acids remained high.

**FIGURE 5:**
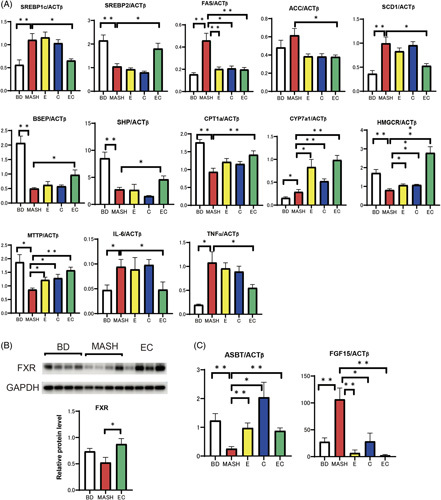
(A) Real-time PCR of the liver samples of mice fed a BD, amylin liver metabolic dysfunction-associated steatohepatitis (amylin MASH) (MASH group), amylin MASH with elobixibat alone (E) and cholestyramine alone (C), or amylin MASH with a combination of EC (n=5–8). (B) FXR protein levels (n=4). (C) Real-time PCR of the ileum samples of mice fed a BD, amylin MASH (MASH group), amylin MASH with elobixibat alone (E) and cholestyramine alone (C), or amylin MASH with a combination of EC (n=5–7). Data are presented as the mean±SE. Significance was determined using Student *t* test. *p<0.05, **p<0.01. Abbreviations: ACC, acetyl-CoA carboxylase; ASBT, apical sodium-dependent bile acid transporter; BD, basal diet; BSEP, bile salt export pump; C, cholestyramine; CYP7a1, cytochrome P450 7A1; E, elobixibat; EC, elobixibat and cholestyramine; FAS, fatty acid synthase; FXR, farnesoid X receptor; GAPDH, glyceraldehyde 3 phosphate dehydrogenase; MASH, metabolic dysfunction-associated steatohepatitis; MTTP, microsomal triglyceride transfer protein; SCD1, stearoyl-CoA desaturase 1; SHP, small heterodimer partner.

In the MASH group, both FITC and LBP levels were more than twice those in the BD group. In group E, FITC levels were twice as high as those in the MASH group. LBP levels were comparable between the MASH and BD groups. Compared with the BD group, intestinal permeability and hyperendotoxemia were increased by elobixibat, while cholestyramine improved intestinal permeability and further reduced endotoxin levels. As shown in Figure 5, IL-6 and TNFα levels exhibited a pattern similar to that of LBP.

Figure 5A shows the results of real-time PCR analysis of the liver samples. Concerning lipid synthesis, SREBf1c expression was suppressed and that of SREBf2 was increased in the EC group. In the MASH group, microsomal triglyceride transfer protein levels decreased to about half of those in the BD group and increased by 1.5-fold those in the EC group.

Compared with that in the BD group, the expression of CYP7a1—a marker of bile acid synthesis—was significantly elevated in the MASH group, and increased by more than 2-fold in groups E and C. In the EC group, it increased more than 3-fold. Compared to the BD group, western blotting showed that the amount of hepatic FXR was significantly lower in the MASH group and significantly higher in the EC group (Fig. 5B). The expression of bile salt export pump and small heterodimer partner was further upregulated in the EC group compared to the MASH group, suggesting that the FXR system in the liver was accelerated in the EC group.

Figure 5C shows real-time PCR of the ileum samples. Compared with those in the BD group, ASBT levels in the ileum decreased to 21% in the MASH group. Compared to that in the MASH group, the expression of ASBT increased 3.7-fold in group E, 3.4-fold in group EC, and 7.9-fold in group C. Conversely, FGF15 expression was upregulated in the MASH group and downregulated in the E, C, and EC groups. This finding indicates that the FXR system was suppressed in the ileum in the EC group.

### Improvement of atherosclerosis

**FIGURE 6:**
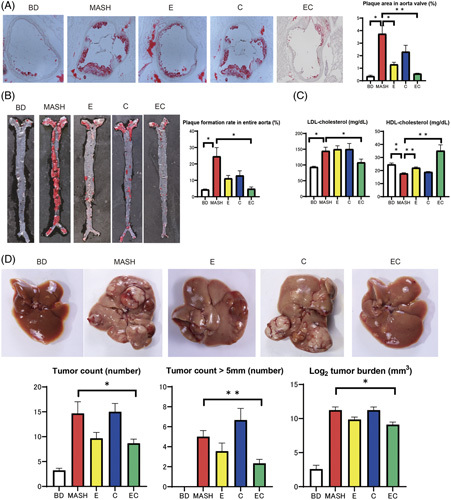
(A) Images of the aortic valve with Oil Red O staining and the percentage area of lesions (Oil Red O positive, n=5–6). The mice were divided into 5 groups according to diet: a BD, amylin liver metabolic dysfunction-associated steatohepatitis (amylin MASH) (MASH group), amylin MASH with elobixibat alone (E) and cholestyramine alone (C), or amylin MASH with a combination of EC. (B) Images of the total aorta with Oil Red O staining and the percentage area of plaque (n=5–6). (C) Serum LDL and HDL cholesterol levels (n=5–7). (D) Images of the livers of diethylnitrosamine-treated mice fed a BD, amylin MASH (MASH group), amylin MASH with elobixibat alone (E) and cholestyramine alone (C), or amylin MASH with a combination of EC. Tumor count, tumor count (>5 mm in diameter), and average log_2_ tumor burden (n=9–10). Data are presented as the mean±SE. Significance was determined using Student *t* test. *p<0.05, **p<0.01. Abbreviations: BD, basal diet; C, cholestyramine; E, elobixibat; EC, elobixibat and cholestyramine; MASH, metabolic dysfunction-associated steatohepatitis.

Supplemental Figure S10, http://links.lww.com/HC9/A624 shows histological images of the liver and lipid profiles in the liver of ApoE (KO) mice fed basal and amylin MASH diets and of those treated with EC. Similar to C57BL/6J mice, amylin MASH–fed ApoE(KO) mice showed significant liver steatosis and fibrosis in the histological analysis (Supplemental Figure S10, http://links.lww.com/HC9/A624 ). Areas of Oil Red O staining were smaller in group E (*p*=0.04) and group EC (*p*=0.007) than in the amylin MASH group. Moreover, areas of Sirius Red staining were smaller in the E (*p*=0.001), C (*p*=0.001), and EC (*p*<0.001) groups than in the MASH group. TG content in the liver was decreased in groups E (*p*=0.04), C (*p*=0.04), and EC (*p*=0.008). For TC and FC content in the liver, no statistically significant change was observed in group C compared to the MASH group, but there was a statistically significant decrease in group E and a further decrease in group EC. The plaque formation rate in the entire aorta was decreased in groups E and C compared with the MASH group and was remarkably decreased in the EC group (Figure 6A). Atherosclerotic lesion formation in the aortic valve was also more pronounced in the MASH group than in the BD group but was significantly improved in the EC group. LDL-cholesterol levels were higher, and HDL cholesterol levels were lower in the MASH group than in the BD group. No change was observed with elobixibat or cholestyramine alone, but cholesterol levels were ameliorated with their combined treatment.

### Inhibitory effect on HCC

Figure 6D shows the tumor counts and burden. Tumor count was significantly higher in the MASH group than in the BD group. Compared with the MASH group, carcinogenesis was suppressed in the EC group. The MASH group had a high tumor burden; however, the combined EC treatment lowered the tumor burden.

## DISCUSSION

In this study, the addition of cholesterol to an HFHF diet resulted in the progression of fatty liver. A cholesterol load of 0.2% or more was sufficient to exacerbate hepatotoxicity and inhibit lipid metabolism parameters. Liver fibrosis increased with increasing cholesterol levels. We propose that lipotoxicity, hyperendotoxemia, and bile acid toxicity were the main mechanisms exacerbating fatty liver in the cholesterol-loaded mouse model (Supplemental Figure S11A, http://links.lww.com/HC9/A625).

We showed that MASH may be caused by suppression of SREBP2 and upregulation of SREBP1c expression. SREBP1c promotes the transcription of genes required for fatty acid synthesis, whereas SREBP2 activates genes required for cholesterol synthesis. Moreover, excessive hepatic cholesterol retention suppresses SREBP2 expression by negative feedback.^20^ SREBP2 is also a negative regulator of SREBP1c.^21^ In this study, the activity of SREBP2 was reduced to suppress the hepatic storage of FC, which was experimentally increased by cholesterol loading but could not be controlled. We consider that this model, with its constant cholesterol uptake, could not limit cholesterol storage. Furthermore, we speculate that the lack of SREBP1c regulation due to the suppression of SREBP2 increased lipid storage. If mice fed an HFHF diet with added cholesterol, SREBP1c expression peaked 1 week after amylin MASH loading, and this change tended to be moderate thereafter. The deceleration of SREBP1c enhancement is likely due to limited TG storage. We believe that a tug-of-war exists between the inhibitory action of SREBP2 and the deceleration of SREBP1c enhancement over time, which complicates interpretation.

To assess whether liver injury is exacerbated by cholesterol load due to cholesterol retention or accumulation of lipids, such as TGs, we knocked down SREBP1c and found that TG levels were reduced and FC levels and bile acid concentration, mainly primary bile acids, were increased in the liver. The decrease in stored fat reduced body and liver weights but worsened liver injury. Thus, TG accumulation may be a protective compensatory response to the lipotoxicity of increased FC levels.

In this study, increased FITC and LBP levels in amylin MASH diet-fed mice suggested possible hyperendotoxemia due to increased intestinal permeability. It has been reported that intestinal permeability is increased by administering CDCA and DCA,^22,23^ which may be involved in intestinal permeability. Previously, HFD mice treated with dextran sulfate sodium showed aggravated hepatosteatosis, fibrosis, and hepatic inflammation, suggesting that barrier dysfunction contributes to disease severity and MASH development. Moreover, portal endotoxin levels were elevated in HFD mice but were significantly elevated in HFD dextran sulfate sodium–administered mice.^24^ MASH has been associated with high blood endotoxin levels.^25^ Another report suggested that hyperleptinemia associated with obesity may increase hepatic KC reactivity to small amounts of endotoxins, inducing MASH development by means of inflammatory cytokines, among other factors.^26^ In this study, the levels of inflammatory cytokines, IL-6 and TNFα, were also elevated in the amylin MASH group, which may be related to hyperendotoxemia.

Bile acids also participate in MASH pathogenesis.^27–29^ Primary bile acids are metabolized by the gut microflora and converted into secondary bile acids, and hydrophobic bile acids are more cytotoxic.^30^ Lithocholic acid, DCA, CDCA, CA, and ursodeoxycholic acid (in increasing order of hydrophobicity)^31^ can cause adverse effects, including mitochondrial dysfunction, endoplasmic reticulum stress, and inflammation.^32^ Additionally, changes in bile acid profiles are highly correlated with intestinal toxemia.^33^

One feedback mechanism through which the healthy hepatic system maintains cholesterol and bile acid homeostasis is mediated by the FXR—a major bile acid–responsive ligand-activated transcription factor that regulates bile acid levels. However, in the MASH model used in this study, hepatic FXR was suppressed despite high serum bile acid levels. Conversely, in the small intestine, the expression of FXR and FGF15, a protein that suppresses bile acid synthesis downstream of FXR signaling, was increased by the HFHF and amylin MASH diets compared to BD. It has been suggested that FXRs in the liver and small intestine behave in opposite manners. Lipopolysaccharides suppress hepatic FXR expression.^34,35^ Hyperendotoxemia in the portal vein may also suppress hepatic FXR expression, resulting in dysregulation of bile acid synthesis. The inhibition of FXR activity by β-muricholic acid, which is unique to mice, may also influence its behavior.^36^ Although a large bile acid pool should normally enhance the FXR system and suppress bile acid synthesis, the suppression of hepatic FXR in MASH model mice due to hyperendotoxemia may result in persistent bile acid synthesis. A constant supply of bile acids and cholesterol would undoubtedly disrupt cholesterol and bile acid homeostasis. In our study, the amylin MASH diet was found to induce all pathological stages of MASLD in C57BL/6J mice when fed for 20 weeks.

To focus on the drugs that could convert FC to bile acids for excretion from the body, we investigated the effect of elobixibat and colestyramine on MASH pathology and found that combined treatment (the EC group) effectively reduced FC retention by reducing the bile acid pool. With colestyramine, the expression of ASBT was upregulated by ~6-fold that in MASH mice. This finding suggests strong, negative feedback between ASBT and bile acid reabsorption. However, this feedback did not result in an effective reduction of FC levels. Although elobixibat alone failed to ameliorate bile acid reabsorption, the combined therapy effectively suppressed the negative feedback mechanism. The reason for elobixibat’s low efficacy is that a single administration increases bile acid levels in stool and intestinal permeability. Bile acids are known to increase intestinal permeability. In high-fat diet mice, a DCA-containing diet increased intestinal permeability by 1.5-fold compared to the control.^22^ Moreover, stool bile acid levels, including levels of secondary bile acids such as DCA, were significantly elevated in that study.^22^ We speculate that ASBT inhibition may increase the portal blood concentration of endotoxins by increasing colonic permeability, thereby accelerating liver injury. This phenomenon, in turn, increases LBP expression and promotes hyperendotoxemia, which may exacerbate liver damage. In combination therapy, cholestyramine can absorb bile acids and ameliorate intestinal permeability, ultimately protecting the liver. The number of pellets and water content of stools increased in the elobixibat group, which was ameliorated by the addition of cholestyramine.

Combination therapy with elobixibat and colestyramine may also be superior in terms of safety as each could compensate for the side effects of the other (Supplemental Figure S11B, http://links.lww.com/HC9/A625). We found that combined administration over the long term was effective in suppressing the development of liver cancer and atherosclerosis, which was associated with the inhibition of MASH. Additionally, the improvement in LDL-cholesterol levels may have contributed to atherosclerosis amelioration.

Efficacy against carcinogenesis may be attributed to the improvement in intrahepatic cholesterol retention and alteration of bile acid profile, in addition to the alleviation of liver fibrosis. One previous study reported a dysregulation of cholesterol metabolism in primary tumors of patients with HCC, with increased mitochondrial cholesterol in HCC cell lines.^37^ The beneficial effects of statins, which inhibit cholesterol biosynthesis, on HCC have been reported in HCC HepG2 cells, rodents with chemically induced HCC, and meta-analyses of clinical trials.^38,39^ Bile acids, a metabolite of cholesterol, are also an independent risk factor for the development of HCC.^40^ According to previous reports, FXR-null mice accumulate bile acids and develop spontaneous HCC in ~90% of cases.^41^ We believe that the combination of EC, in addition to improving fibrosis in MASH, lowered FC in the liver and had a corrective effect on bile acids, thereby preventing carcinogenesis.

Our study has some limitations. First, we used a single dose (based on a previous report)^12^ of elobixibat and colestyramine to compare their therapeutic effect on MASH. Second, the NAS and fibrosis stages of the liver samples were not scored according to the method of Kleiner et al.^42–46^ Ideally, an expert in mouse histopathology would have conducted the evaluations, but we have no such expert at our facility. Finally, we have not been able to prove a causal relationship between MASH progression and changes in factors that regulate lipids and bile acids, including SREBP2, CYP7a1, and microsomal triglyceride transfer protein. To examine the effects of each factor, it is necessary to conduct detailed studies that involve gene knockdown or the addition of external factors that promote the effects of each factor, which should be performed in the future.

In conclusion, FC levels in the liver can progress to fatty liver disease. To reduce FC levels, combination therapy with EC was effective in a mouse model of MASH. Our findings suggest that combination therapy could be an effective and safe treatment for MASH.

## Supplementary Material

## References

[1] Rinella ME Lazarus JV Ratziu V Francque SM Sanyal AJ Kanwal F. A multi-society Delphi consensus statement on new fatty liver disease nomenclature. Ann Hepatol. 2023:101133. doi:10.1016/j.aohep.2023.101133. 37364816

[2] Rinella ME Lazarus JV Ratziu V Francque SM Sanyal AJ Kanwal F. A multi-society Delphi consensus statement on new fatty liver disease nomenclature. J Hepatol. 2023. doi:10.1016/j.jhep.2023.06.003 37983810

[3] Rinella ME Lazarus JV Ratziu V Francque SM Sanyal AJ Kanwal F. A multi-society Delphi consensus statement on new fatty liver disease nomenclature. Hepatology. 2023. Publish Ahead of Print. doi:10.1097/HEP.0000000000000520 37983810

[4] Day CP. Natural history of NAFLD: Remarkably benign in the absence of cirrhosis. Gastroenterology. 2005;129:375–8. 16012969 10.1053/j.gastro.2005.05.041

[5] Jump DB Depner CM Tripathy S Lytle KA. Impact of dietary fat on the development of non-alcoholic fatty liver disease in Ldlr-/- mice. Proc Nutr Soc. 2016;75:1–9. 26282529 10.1017/S002966511500244X PMC4720541

[6] Noureddin M Zelber‐Sagi S Wilkens LR Porcel J Boushey CJ Le Marchand L. Diet associations with nonalcoholic fatty liver disease in an ethnically diverse population: The multiethnic cohort. Hepatology. 2020;71:1940–52. 31553803 10.1002/hep.30967 PMC7093243

[7] Wouters K van Bilsen M van Gorp PJ Bieghs V Lütjohann D Kerksiek A. Intrahepatic cholesterol influences progression, inhibition and reversal of non-alcoholic steatohepatitis in hyperlipidemic mice. FEBS Lett. 2010;584:1001–05. 20114046 10.1016/j.febslet.2010.01.046

[8] Yamaguchi K Yang L McCall S Huang J Yu XX Pandey SK. Inhibiting triglyceride synthesis improves hepatic steatosis but exacerbates liver damage and fibrosis in obese mice with nonalcoholic steatohepatitis. Hepatology. 2007;45:1366–74. 17476695 10.1002/hep.21655

[9] Ferslew BC Xie G Johnston CK Su M Stewart PW Jia W. Altered bile acid metabolome in patients with nonalcoholic steatohepatitis. Dig Dis Sci. 2015;60:3318–28. 26138654 10.1007/s10620-015-3776-8 PMC4864493

[10] Chavez-Talavera O Tailleux A Lefebvre P Staels B. Bile acid control of metabolism and inflammation in obesity, type 2 diabetes, dyslipidemia, and nonalcoholic fatty liver disease. Gastroenterology. 2017;152:1679–94.e1673. 28214524 10.1053/j.gastro.2017.01.055

[11] Xiao L Pan G. An important intestinal transporter that regulates the enterohepatic circulation of bile acids and cholesterol homeostasis: The apical sodium-dependent bile acid transporter (SLC10A2/ASBT). Clin Res Hepatol Gastroenterol. 2017;41:509–15. 28336180 10.1016/j.clinre.2017.02.001

[12] Rao A Kosters A Mells JE Zhang W Setchell KDR Amanso AM. Inhibition of ileal bile acid uptake protects against nonalcoholic fatty liver disease in high-fat diet-fed mice. Sci Transl Med. 2016;8:357ra122. 10.1126/scitranslmed.aaf4823 PMC5056562 27655848

[13] Barkun AN Love J Gould M Pluta H Steinhart H. Bile acid malabsorption in chronic diarrhea: Pathophysiology and treatment. Can J Gastroenterol. 2013;27:653–9. 24199211 10.1155/2013/485631 PMC3816948

[14] Taniai M Hashimoto E Tobari M Yatsuji S Haruta I Tokushige K. Treatment of nonalcoholic steatohepatitis with colestimide. Hepatol Res. 2009;39:685–93. 19473432 10.1111/j.1872-034X.2009.00507.x

[15] Le TA Chen J Changchien C Peterson MR Kono Y Patton H. Effect of colesevelam on liver fat quantified by magnetic resonance in nonalcoholic steatohepatitis: A randomized controlled trial. Hepatology. 2012;56:922–32. 22431131 10.1002/hep.25731 PMC3400720

[16] Uehara T Ainslie GR Kutanzi K Pogribny IP Muskhelishvili L Izawa T. Molecular mechanisms of fibrosis-associated promotion of liver carcinogenesis. Toxicol Sci. 2013;132:53–63. 23288052 10.1093/toxsci/kfs342 PMC3576012

[17] Fuchs BC Hoshida Y Fujii T Wei L Yamada S Lauwers GY. Epidermal growth factor receptor inhibition attenuates liver fibrosis and development of hepatocellular carcinoma. Hepatology. 2014;59:1577–90. 24677197 10.1002/hep.26898 PMC4086837

[18] Li S Ghoshal S Sojoodi M Arora G Masia R Erstad DJ. Pioglitazone reduces hepatocellular carcinoma development in two rodent models of cirrhosis. J Gastrointest Surg. 2019;23:101–11. 30367397 10.1007/s11605-018-4004-6 PMC6328630

[19] Verna L Whysner J Williams GM. N-nitrosodiethylamine mechanistic data and risk assessment: Bioactivation, DNA-adduct formation, mutagenicity, and tumor initiation. Pharmacol Ther. 1996;71:57–81. 8910949 10.1016/0163-7258(96)00062-9

[20] Shimano H. SREBPs: Physiology and pathophysiology of the SREBP family. FEBS J. 2009;276:616–21. 19143830 10.1111/j.1742-4658.2008.06806.x

[21] Horie T Nishino T Baba O Kuwabara Y Nakao T Nishiga M. MicroRNA-33 regulates sterol regulatory element-binding protein 1 expression in mice. Nat Commun. 2013;4:2883. 24300912 10.1038/ncomms3883 PMC3863899

[22] Stenman LK Holma R Eggert A Korpela R. A novel mechanism for gut barrier dysfunction by dietary fat: Epithelial disruption by hydrophobic bile acids. Am J Physiol Gastrointest Liver Physiol. 2013;304:G227–34. 23203158 10.1152/ajpgi.00267.2012

[23] Song M Ye J Zhang F Su H Yang X He H. Chenodeoxycholic acid (CDCA) protects against the lipopolysaccharide-induced impairment of the intestinal epithelial barrier function via the FXR-MLCK pathway. J Agric Food Chem. 2019;67:8868–74. 31319027 10.1021/acs.jafc.9b03173

[24] Gäbele E Dostert K Hofmann C Wiest R Schölmerich J Hellerbrand C. DSS induced colitis increases portal LPS levels and enhances hepatic inflammation and fibrogenesis in experimental NASH. J Hepatol. 2011;55:1391–9. 21703208 10.1016/j.jhep.2011.02.035

[25] Harte AL da Silva NF Creely SJ McGee KC Billyard T Youssef-Elabd EM. Elevated endotoxin levels in non-alcoholic fatty liver disease. J Inflamm (Lond). 2010;7:15. 20353583 10.1186/1476-9255-7-15 PMC2873499

[26] Imajo K Fujita K Yoneda M Nozaki Y Ogawa Y Shinohara Y. Hyperresponsivity to low-dose endotoxin during progression to nonalcoholic steatohepatitis is regulated by leptin-mediated signaling. Cell Metab. 2012;16:44–54. 22768838 10.1016/j.cmet.2012.05.012

[27] Gottlieb A Bechmann L Canbay A. The presence and severity of nonalcoholic steatohepatitis is associated with specific changes in circulating bile acids. Ann Hepatol. 2018;17:340–1. 29735794 10.5604/01.3001.0011.7378

[28] Aranha MM Cortez-Pinto H Costa A da Silva IBM Camilo ME de Moura MC. Bile acid levels are increased in the liver of patients with steatohepatitis. Eur J Gastroenterol Hepatol. 2008;20:519–25. 18467911 10.1097/MEG.0b013e3282f4710a

[29] Kalhan SC Guo L Edmison J Dasarathy S McCullough AJ Hanson RW. Plasma metabolomic profile in nonalcoholic fatty liver disease. Metabolism. 2011;60:404–13. 20423748 10.1016/j.metabol.2010.03.006 PMC2950914

[30] Singh M Singh A Kundu S Bansal S Bajaj A. Deciphering the role of charge, hydration, and hydrophobicity for cytotoxic activities and membrane interactions of bile acid based facial amphiphiles. Biochim Biophys Acta. 2013;1828:1926–37. 23590996 10.1016/j.bbamem.2013.04.003

[31] Perez MJ Briz O. Bile-acid-induced cell injury and protection. World J Gastroenterol. 2009;15:1677–89. 19360911 10.3748/wjg.15.1677 PMC2668773

[32] Marin JJ Macias RI Briz O Banales JM Monte MJ. Bile acids in physiology, pathology and pharmacology. Curr Drug Metab. 2015;17:4–29. 26526836 10.2174/1389200216666151103115454

[33] Gómez-Zorita S Aguirre L Milton-Laskibar I Fernández-Quintela A Trepiana J Kajarabille N. Relationship between changes in microbiota and liver steatosis induced by high-fat feeding-a review of rodent models. Nutrients. 2019;11:2156. 31505802 10.3390/nu11092156 PMC6770892

[34] Kim MS Shigenaga J Moser A Feingold K Grunfeld C. Repression of farnesoid X receptor during the acute phase response. J Biol Chem. 2003;278:8988–95. 12519762 10.1074/jbc.M212633200

[35] Beigneux AP Moser AH Shigenaga JK Grunfeld C Feingold KR. The acute phase response is associated with retinoid X receptor repression in rodent liver. J Biol Chem. 2000;275:16390–9. 10747970 10.1074/jbc.M000953200

[36] Puri P Daita K Joyce A Mirshahi F Santhekadur PK Cazanave S. The presence and severity of nonalcoholic steatohepatitis is associated with specific changes in circulating bile acids. Hepatology. 2018;67:534–48. 28696585 10.1002/hep.29359 PMC5764808

[37] Montero J Morales A Llacuna L Lluis JM Terrones O Basañez G. Mitochondrial cholesterol contributes to chemotherapy resistance in hepatocellular carcinoma. Cancer Res. 2008;68:5246–56. 18593925 10.1158/0008-5472.CAN-07-6161

[38] Ridruejo E Romero-Caimi G Obregon MJ Kleiman de Pisarev D Alvarez L. Potential molecular targets of statins in the prevention of hepatocarcinogenesis. Ann Hepatol. 2018;17:490–500. 29735800 10.5604/01.3001.0011.7394

[39] Facciorusso A Abd El Aziz MA Singh S Pusceddu S Milione M Giacomelli L. Statin use decreases the incidence of hepatocellular carcinoma: An updated meta-analysis. Cancers (Basel). 2020;12:874. 32260179 10.3390/cancers12040874 PMC7225931

[40] Wang H Shang X Wan X Xiang X Mao Q Deng G. Increased hepatocellular carcinoma risk in chronic hepatitis B patients with persistently elevated serum total bile acid: A retrospective cohort study. Sci Rep. 2016;6:38180. 27905528 10.1038/srep38180 PMC5131293

[41] Kim I Morimura K Shah Y Yang Q Ward JM Gonzalez FJ. Spontaneous hepatocarcinogenesis in farnesoid X receptor-null mice. Carcinogenesis. 2007;28:940–6. 17183066 10.1093/carcin/bgl249 PMC1858639

[42] Matye DJ Li Y Chen C Chao X Wang H Ni H. Gut-restricted apical sodium-dependent bile acid transporter inhibitor attenuates alcohol-induced liver steatosis and injury in mice. Alcohol Clin Exp Res. 2021;45:1188–99. 33885179 10.1111/acer.14619 PMC8717856

[43] Matsui M Fukunishi S Nakano T Ueno T Higuchi K Asai A. Ileal bile acid transporter inhibitor improves hepatic steatosis by ameliorating gut microbiota dysbiosis in NAFLD model mice. mBio. 2021;12:e0115521. 34225483 10.1128/mBio.01155-21 PMC8406289

[44] Kleiner DE Brunt EM Van Natta M Behling C Contos MJ Cummings OW. Design and validation of a histological scoring system for nonalcoholic fatty liver disease. Hepatology. 2005;41:1313–21. 15915461 10.1002/hep.20701

[45] Zhu A Chen J Wu P Luo M Zeng Y Liu Y. Cationic polystyrene resolves nonalcoholic steatohepatitis, obesity, and metabolic disorders by promoting eubiosis of gut microbiota and decreasing endotoxemia. Diabetes. 2017;66:2137–43. 28446519 10.2337/db17-0070 PMC5521855

[46] Matsumoto K Yokoyama S. Gene expression analysis on the liver of cholestyramine-treated type 2 diabetic model mice. Biomed Pharmacother. 2010;64:373–8. 20347570 10.1016/j.biopha.2010.02.008

