# Supplementary figures and images for: Combined, elobixibat, and colestyramine reduced cholesterol toxicity in a mouse model of metabolic dysfunction-associated steatotic liver disease
Source: Hepatol Commun. 2023 Oct 31;7(11):e0285. doi: 10.1097/HC9.0000000000000285 (PMC10617934; doi:10.1097/HC9.0000000000000285)

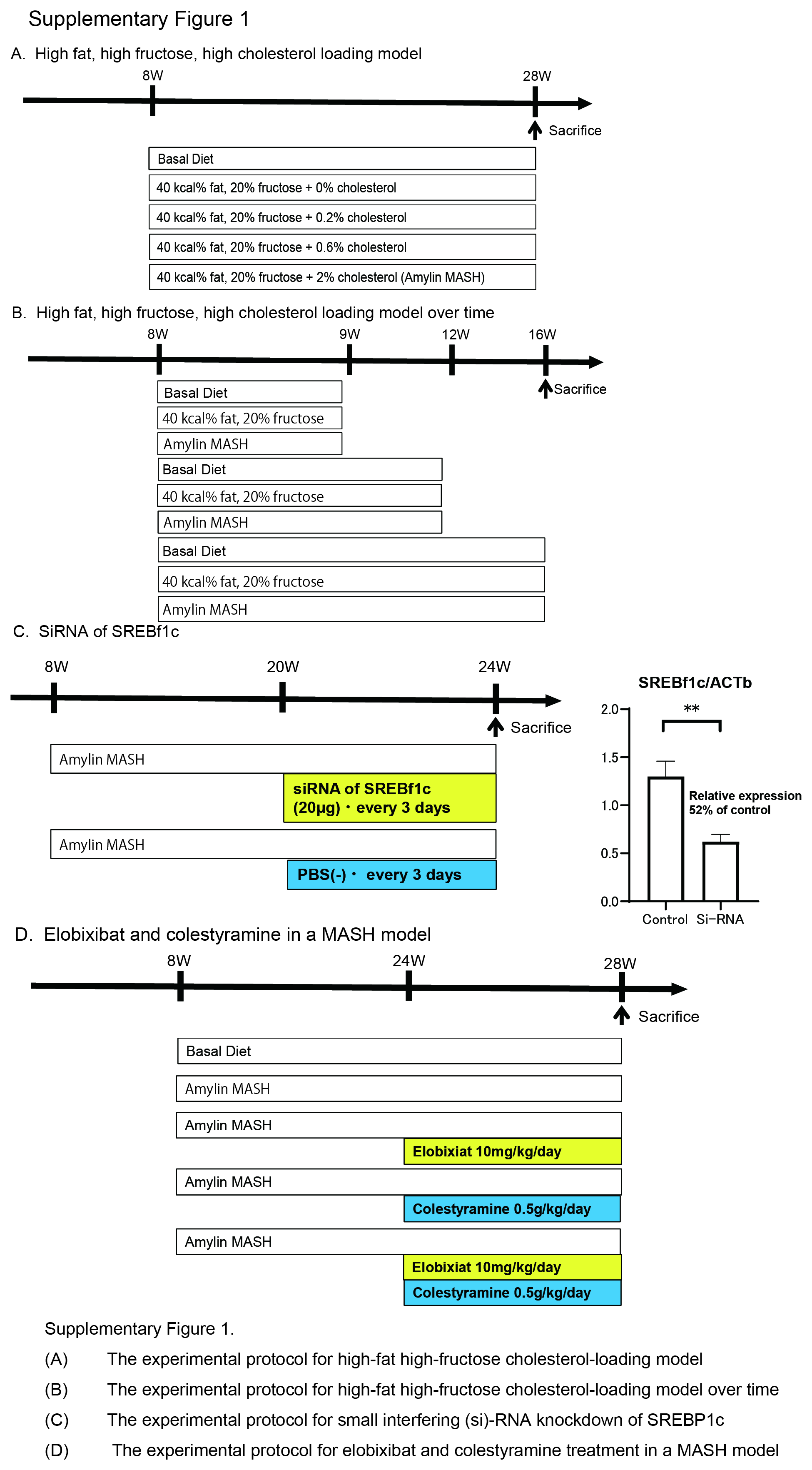

Supplement: Supplementary file 1 [file hc9-7-e0285-s001.tif]

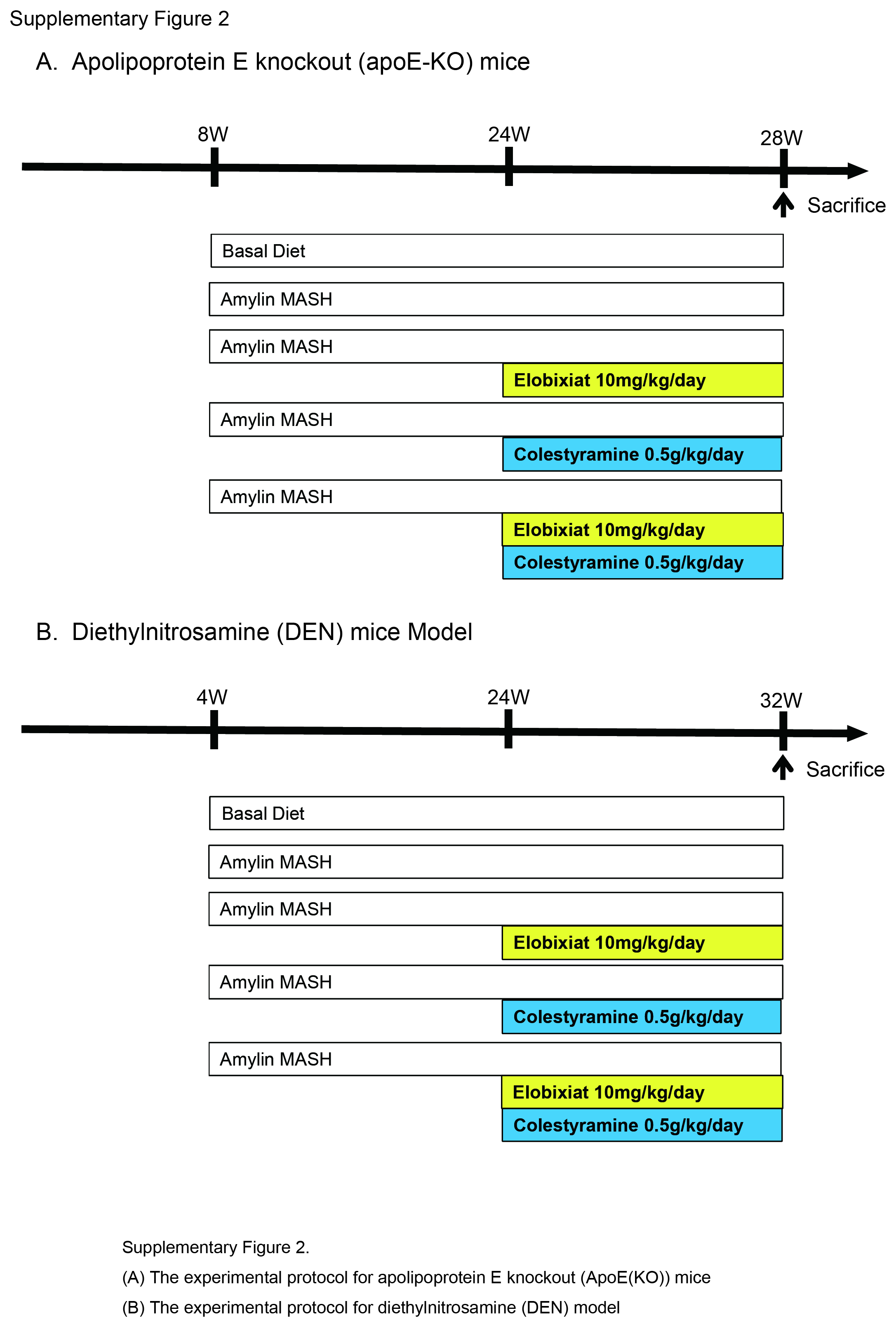

Supplement: Supplementary file 2 [file hc9-7-e0285-s002.tif]

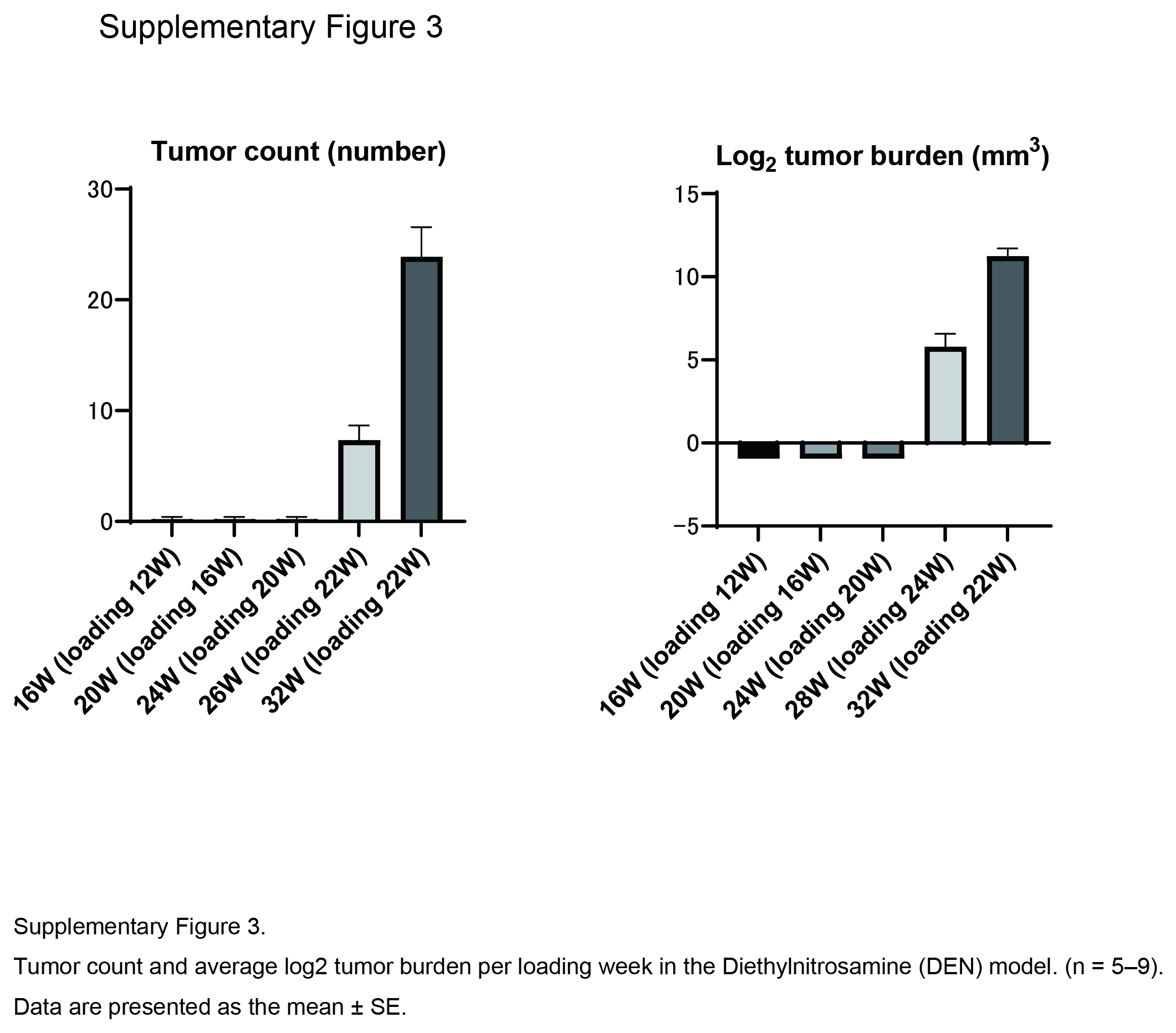

Supplement: Supplementary file 3 [file hc9-7-e0285-s003.tif]

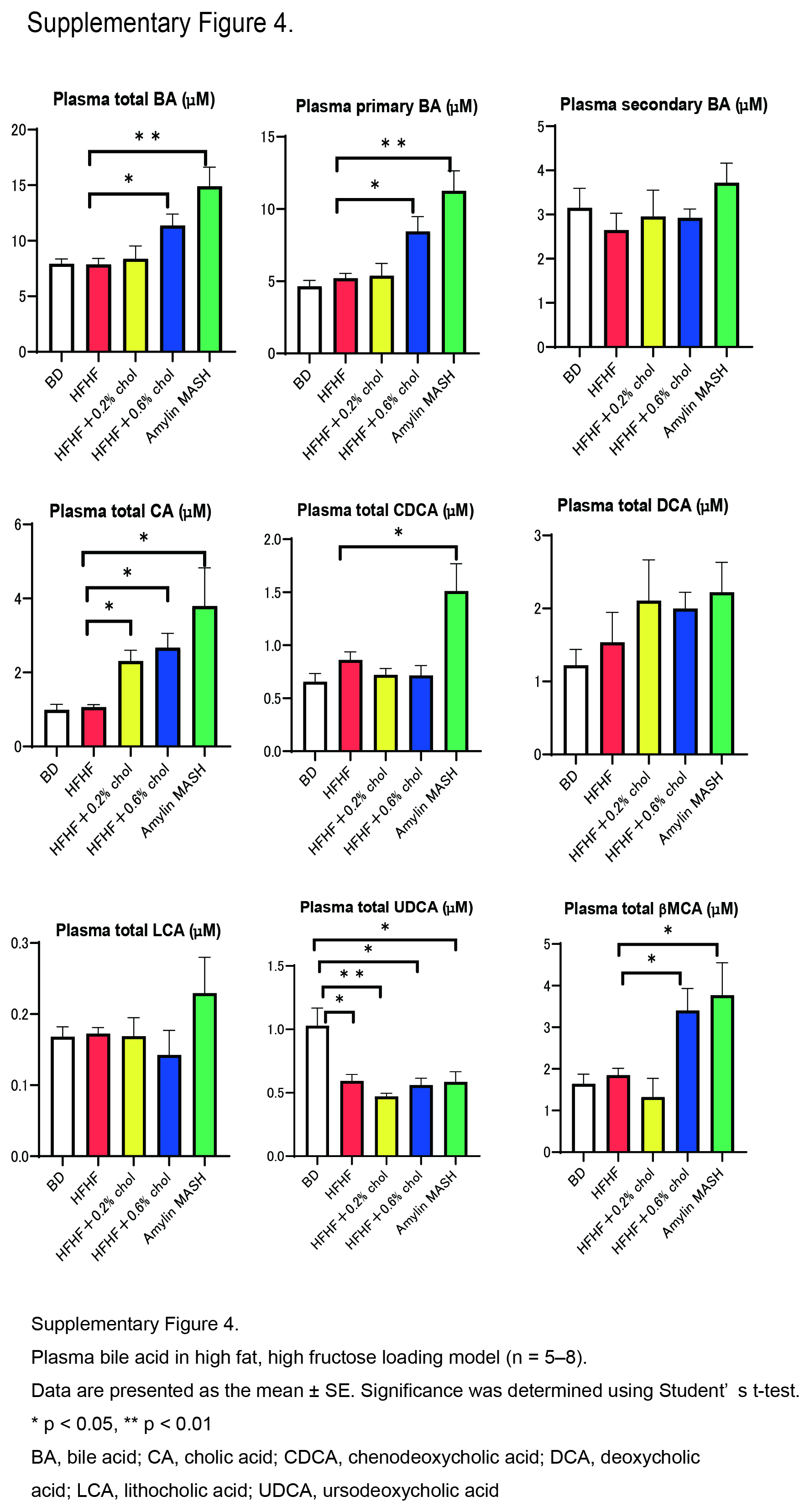

Supplement: Supplementary file 4 [file hc9-7-e0285-s004.tif]

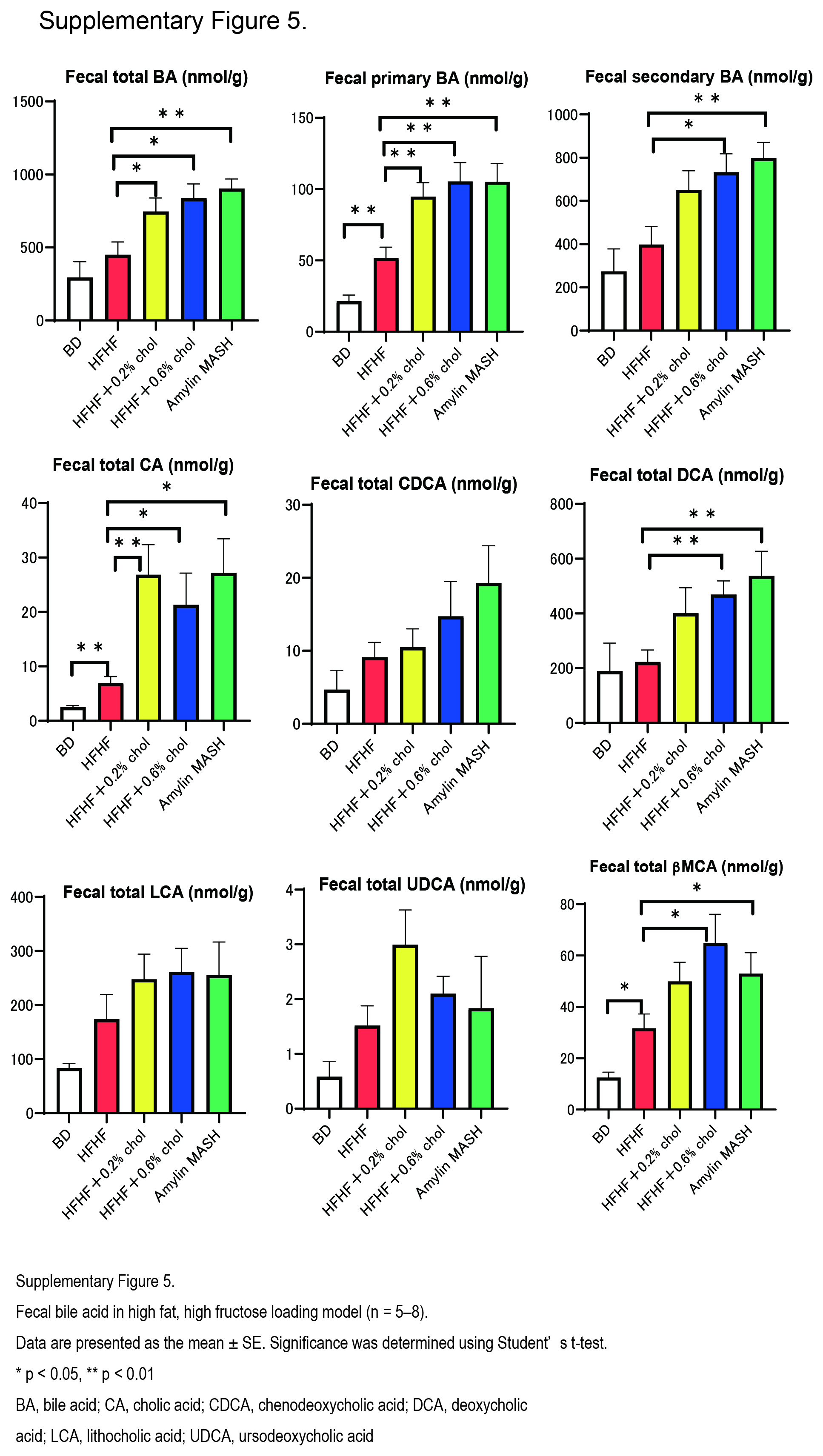

Supplement: Supplementary file 5 [file hc9-7-e0285-s005.tif]

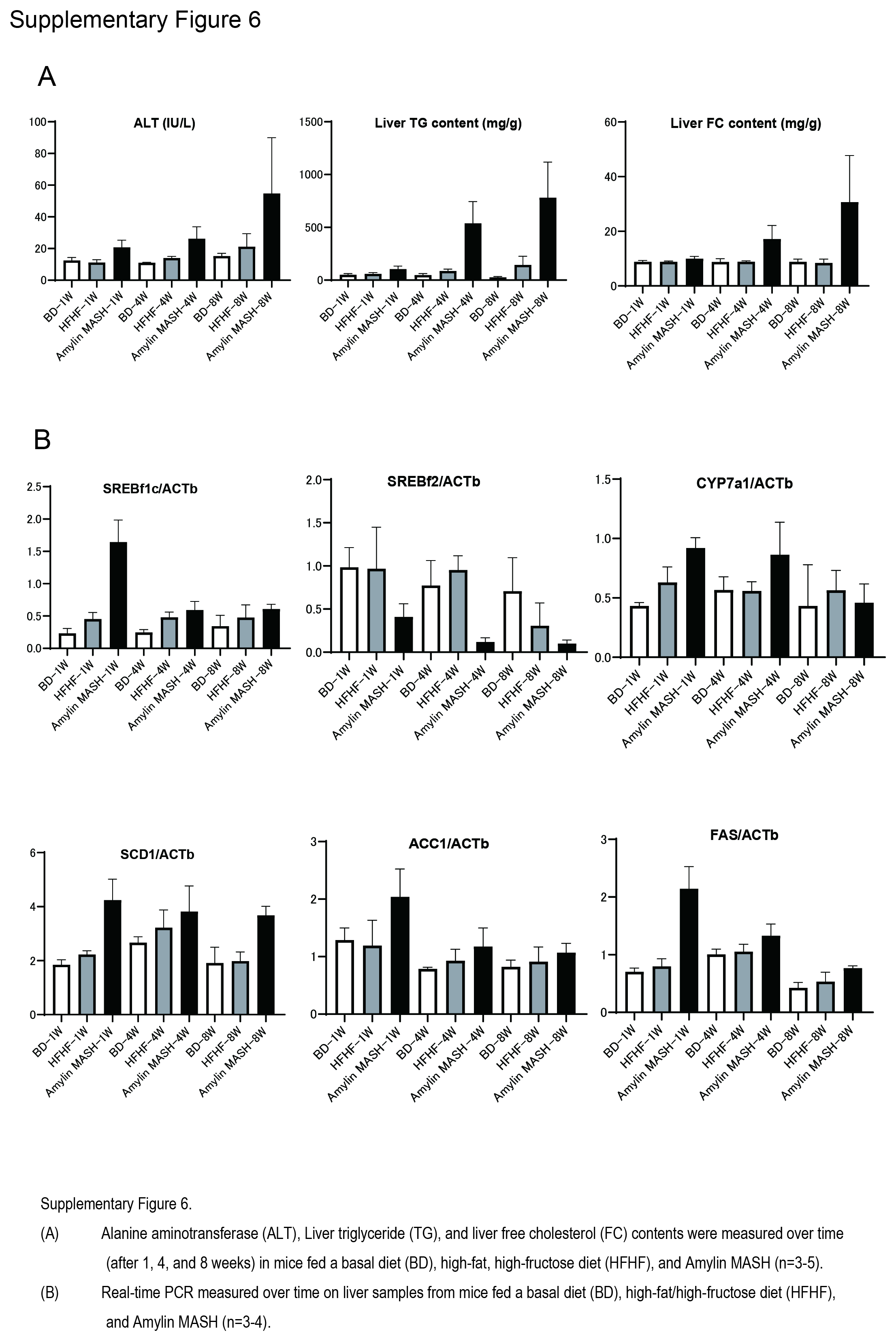

Supplement: Supplementary file 6 [file hc9-7-e0285-s006.tif]

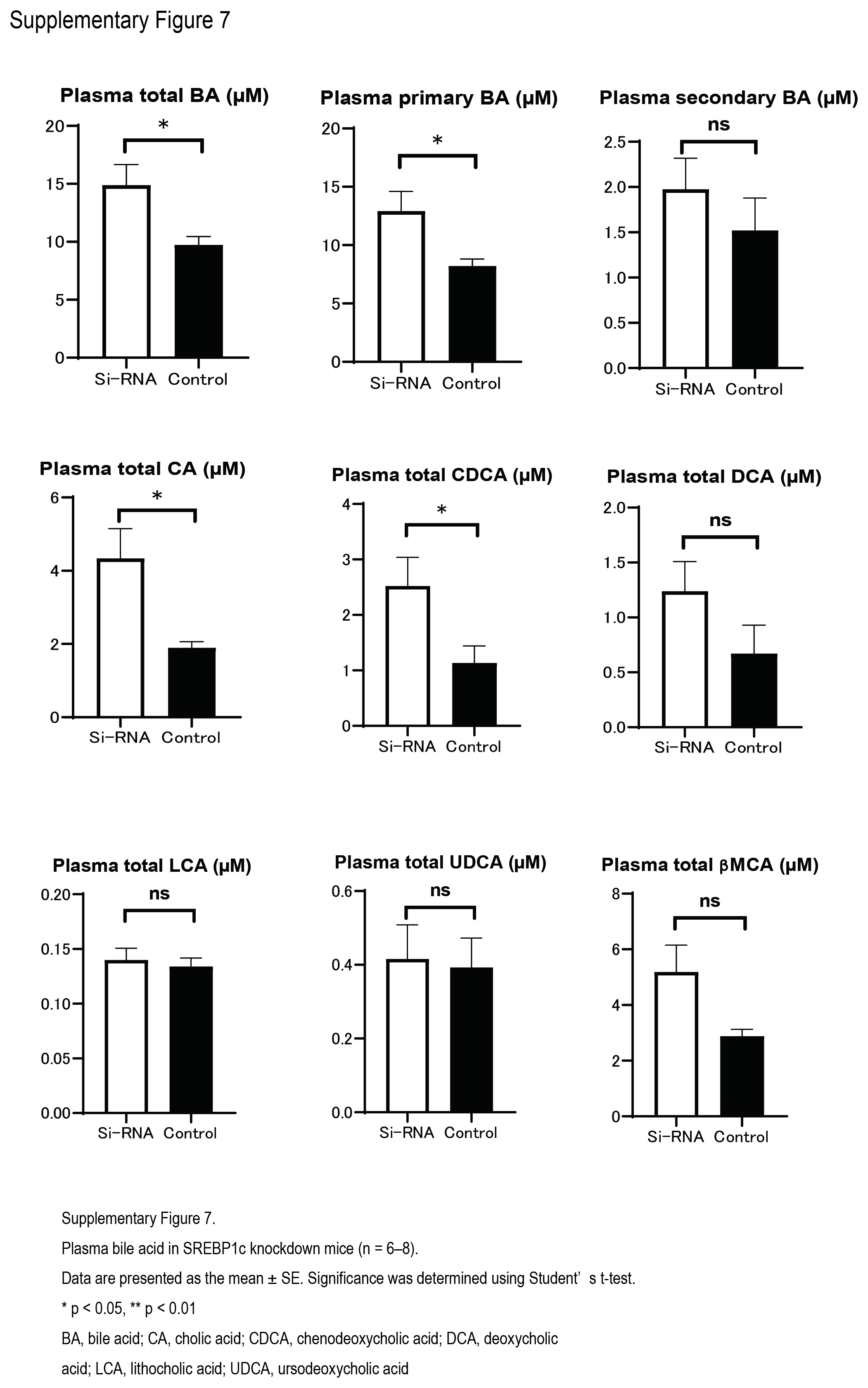

Supplement: Supplementary file 7 [file hc9-7-e0285-s007.tif]

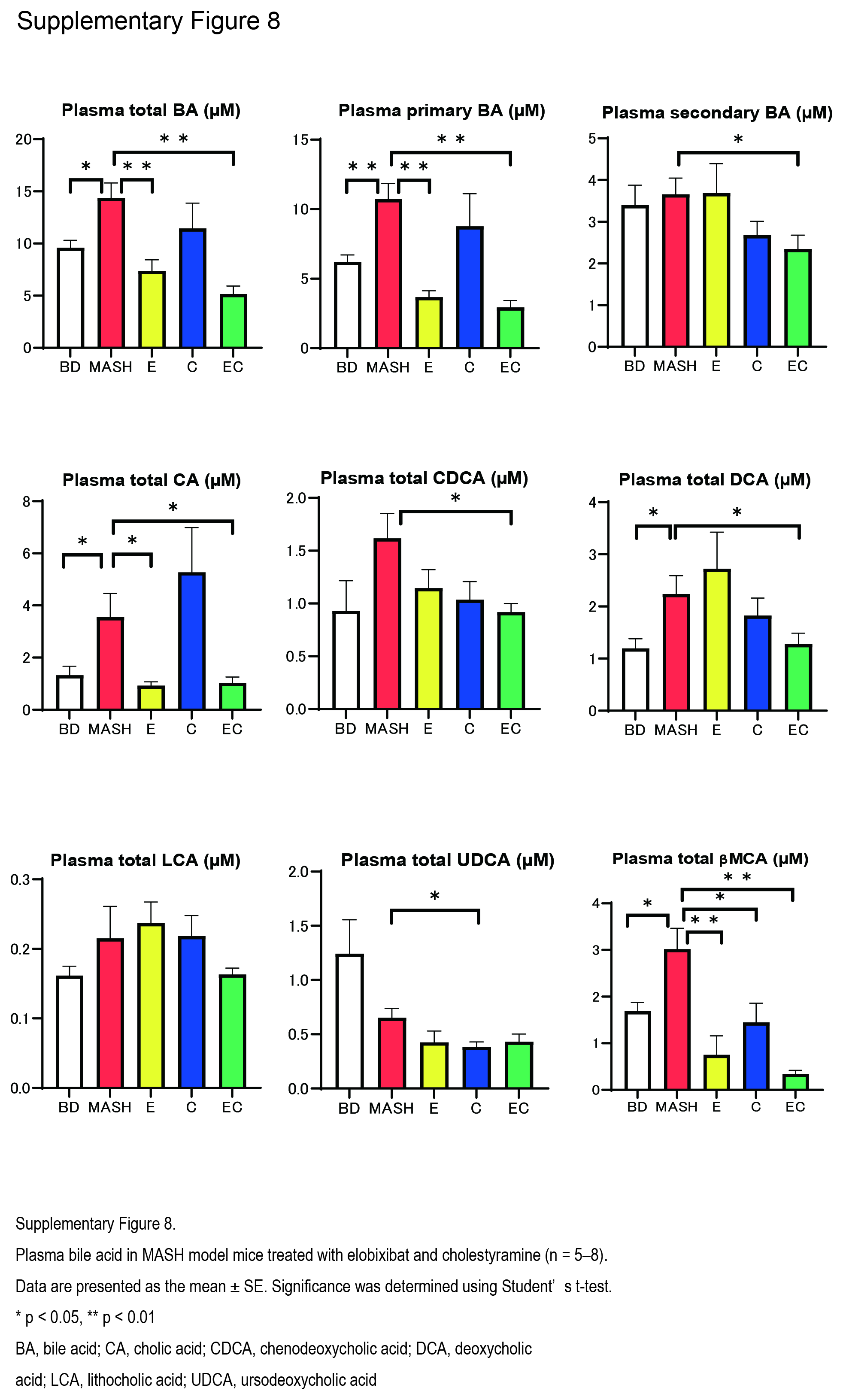

Supplement: Supplementary file 8 [file hc9-7-e0285-s008.tif]

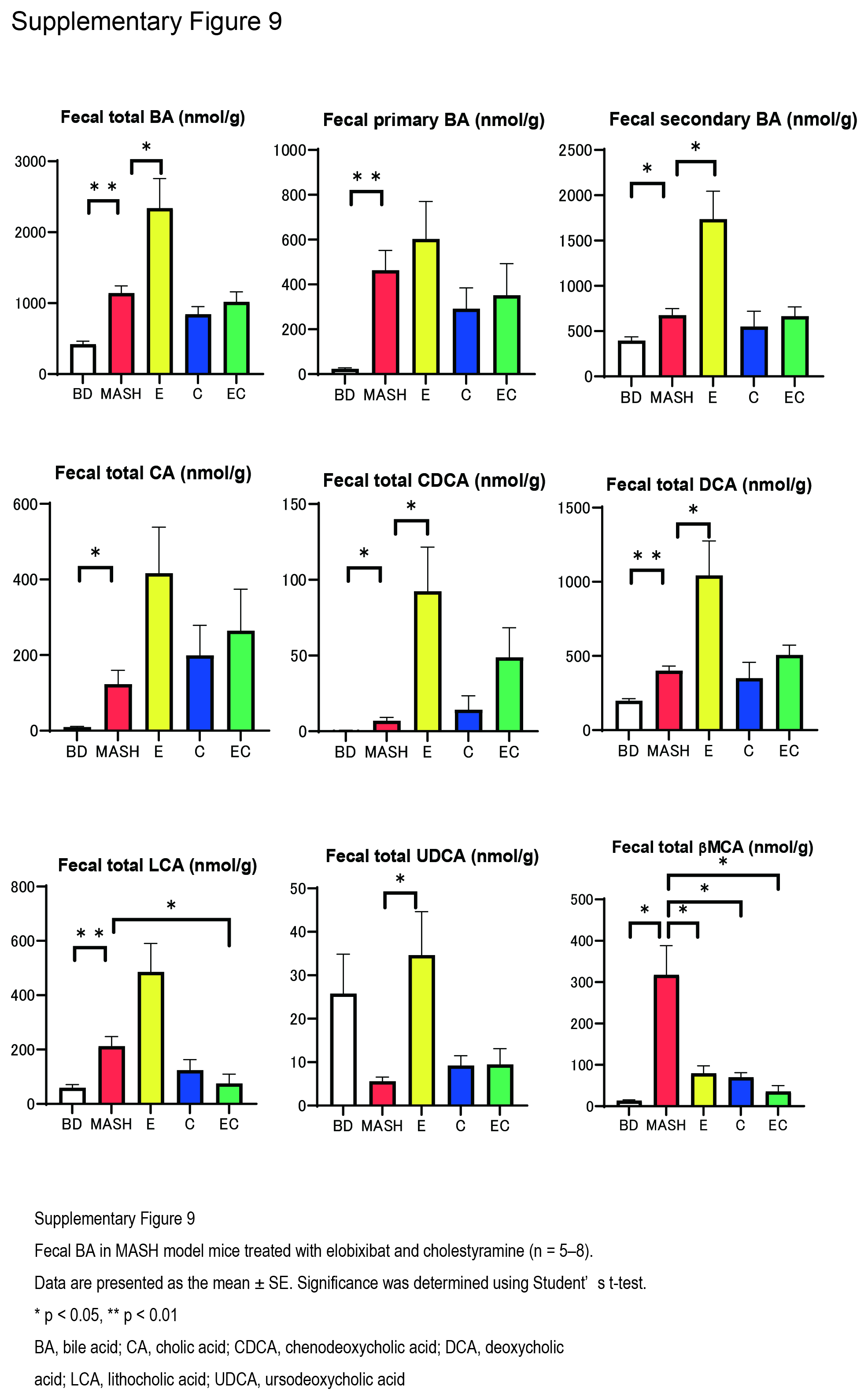

Supplement: Supplementary file 9 [file hc9-7-e0285-s009.tif]

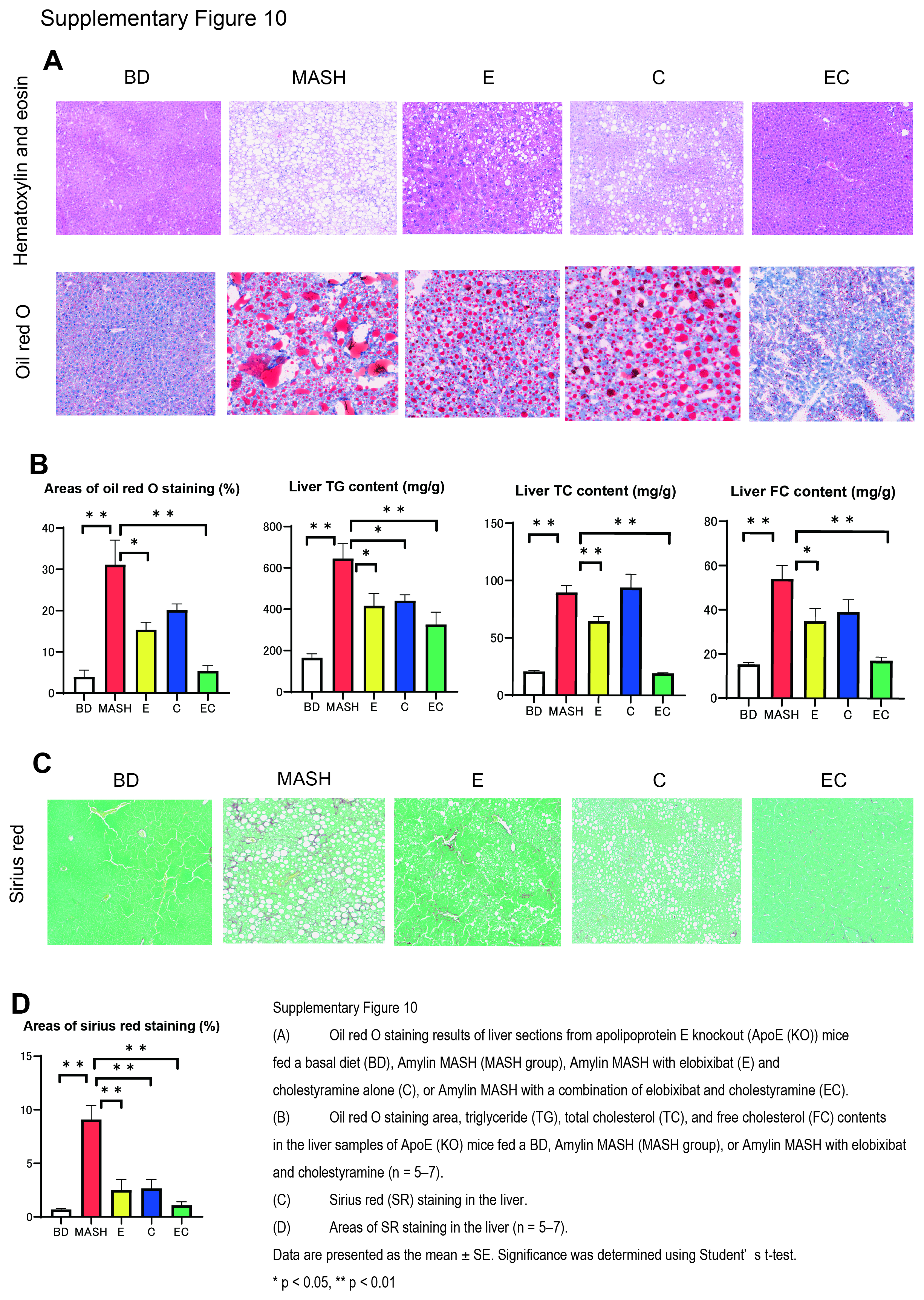

Supplement: Supplementary file 10 [file hc9-7-e0285-s010.tif]

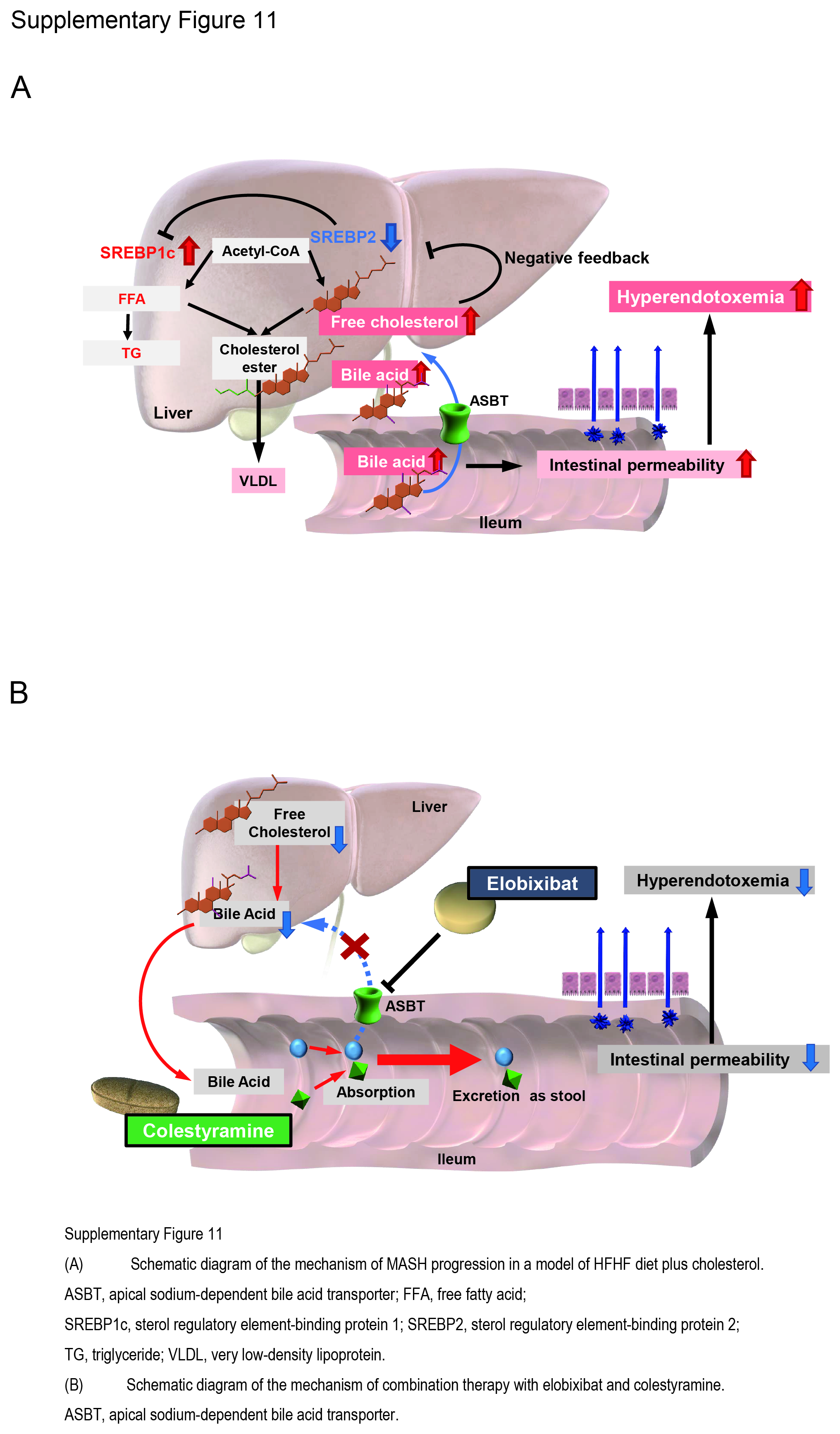

Supplement: Supplementary file 11 [file hc9-7-e0285-s011.tif]
