# Supplementary material for: Combined, elobixibat, and colestyramine reduced cholesterol toxicity in a mouse model of metabolic dysfunction-associated steatotic liver disease
Source: Hepatol Commun. 2023 Oct 31;7(11):e0285. doi: 10.1097/HC9.0000000000000285 (PMC10617934; doi:10.1097/HC9.0000000000000285)
Supplement: Supplementary file 12 [file hc9-7-e0285-s012.docx]

**Supplementary table 1. Characteristics of SREBP1c knockdown mice.**

| Parameters | Si-RNA mice | control | p |
| --- | --- | --- | --- |
| Body weight (g) | 35.9 ± 0.6 | 38.9 ± 0.7 | 0.008 |
| Liver weight (g) | 3.2 ± 0.2 | 3.8 ± 0.2 | 0.016 |
| Epididymal adipose tissue weight (g) | 1.7 ± 0.1 | 1.9 ± 0.1 | 0.20 |
| AST (IU/L) | 170.6 ± 15.7 | 122.0 ± 14.1 | 0.048 |
| ALT (IU/L) | 244.4 ± 22.1 | 157.3 ± 23.3 | 0.024 |
| Total cholesterol (mg/dL) | 246.0 ± 4.2 | 205.5 ± 12.4 | 0.021 |
| Free fatty acid (uEQ/L) | 849.2 ± 67.2 | 795.5 ± 76.1 | 0.61 |

Data are presented as the mean ± SE (n = 6–7). Significance was determined using Student’s *t*-test.

ALT, alanine aminotransferase; AST, aspartate aminotransferase; siRNA, small interfering RNA; SREBP1c, sterol regulatory element-binding protein 1C.
