# Supplementary material for: Combined, elobixibat, and colestyramine reduced cholesterol toxicity in a mouse model of metabolic dysfunction-associated steatotic liver disease
Source: Hepatol Commun. 2023 Oct 31;7(11):e0285. doi: 10.1097/HC9.0000000000000285 (PMC10617934; doi:10.1097/HC9.0000000000000285)
Supplement: Supplementary file 13 [file hc9-7-e0285-s013.docx]

**Supplementary Materials**

**Combined, elobixibat and colestyramine reduced cholesterol toxicity in a mouse model of metabolic dysfunction-associated steatotic liver disease**

Michihiro Iwaki^1^, Takaomi Kessoku^2^, Kosuke Tanaka^1^, Anna Ozaki^1^, Yuki Kasai^1^, Takashi Kobayashi^1^, Yasushi Honda^3^, Yuji Ogawa^4^, Kento Imajo^5^, Haruki Usuda^6^, Koichiro Wada^6^, Noritoshi Kobayashi^7^, Satoru Saito^1^, Atsushi Nakajima^1^, Masato Yoneda^1^.

^1^ Department of Gastroenterology and Hepatology, Yokohama City University Graduate School of Medicine, 3-9 Fukuura, Kanazawa-ku, Yokohama, 236-0004, Japan

^2^ Department of Palliative Medicine, International University Health and Welfare, Narita Hospital, 852 Hatakeda, Narita, 286-8520, Japan

^3^ Department of Internal Medicine, Asakura Hospital, 5-81-12 Shimonagaya, Konan-ku, Yokohama, 233-0016, Japan

^4^ Department of Gastroenterology, National Hospital Organization Yokohama Medical Center, 3-60-2 Harajuku, Totsuka-ku, Yokohama, 245-8575, Japan

^5^ Department of Gastroenterology, Shinyurigaoka General Hospital, 255 Furusawa Asao-ku, Kawasaki, 215-0026, Japan

^6^ Department of Pharmacology, Shimane University Faculty of Medicine, Shimane, 693-8501, Japan

^7^ Department of Oncology, Yokohama City University Graduate School of Medicine, 3-9 Fukuura, Kanazawa-ku, Yokohama, 236-0004, Japan

*Corresponding author:

Masato Yoneda

Department of Gastroenterology and Hepatology, Yokohama City University Graduate School of Medicine, 3-9 Fukuura, Kanazawa-ku, Yokohama 236-0004, Japan

Tel.: +81-45-787-2640; Fax: +81-45-784-3546;

E-mail: [yoneda@yokohama-cu.ac.jp](mailto:yoneda@yokohama-cu.ac.jp)

**APPENDIX 1**

**Sample collection and serological examination**

All blood samples—collected from the portal vein—were treated with heparin, and plasma was isolated by centrifugation at 1000 × *g* for 15 min at 4 °C. The samples were maintained at −80 °C until use. Serum levels of aspartate aminotransferase (AST), alanine aminotransferase (ALT), total cholesterol, fasting plasma glucose, and free fatty acids (FFAs) were measured at a local laboratory (SRL, Tokyo, Japan). Insulin was assessed using an ELISA kit (Morinaga, Tokyo, Japan).

The homeostasis model assessment of insulin resistance (HOMA-IR) was calculated as:

HOMA-IR = insulin (µU/mL) × fasting plasma glucose (mg/dL) / 405

Lipopolysaccharide binding protein (LBP) was measured using an ELISA kit (Hycult Biotech, Uden, Netherlands).

Fluorescein isothiocyanate (FITC)—a fluorescently labeled polymer of anhydroglucose—was purchased from Sigma-Aldrich (St. Louis, MO, USA). It is a large molecule (3–5 kDa) that cannot cross the epithelial barrier under normal conditions.(1) However, disruption of tight junctions, as occurs during intestinal inflammation, allows FITC-dextran to cross the epithelial barrier, making it a useful marker of intestinal permeability.

**APPENDIX 2**

**Liver histology**

For histological examination, portions of liver lobes from each mouse were fixed in 10% formalin and embedded in paraffin. Paraffin-embedded sections were stained with hematoxylin and eosin (H&E) and Sirius red (SR). For lipid staining, frozen sections were stained with Oil Red O. The areas stained by Oil Red O and Sirius red were quantified from five random fields of each liver section with Image J.(2) Lipids were extracted from liver tissue (50 mg) using chloroform:methanol (2:1), as described by Folch et al.(3) Triglycerides, total cholesterol, and free cholesterol levels in the liver were measured using colorimetric assays (FUJIFILM Wako Pure Chemical Corporation, Tokyo, Japan).

**APPENDIX 3**

**Evaluation of atherosclerosis**

Atherosclerotic lesion formation in the aortic root and surface of the aorta were quantitatively analyzed as previously described.(4,5)

A fixed heart was cut in a plane parallel to the atrial appendages, and the upper part was embedded in an optimal cutting temperature compound (Sakura Finetec, Tokyo, Japan).

Cryostat sections were cut from the left ventricular outflow tract until the valve cusps were exposed. Thereafter, 45 serial 8-μm-thick sections were prepared. Nine sections (every five, each separated by 40 μm) were stained with Oil Red O and counterstained with hematoxylin. The Oil Red O-stained lesion area was discriminated manually from the unstained regions of the photomicrograph images using Photoshop Elements software (v.9.0.3; Adobe Systems, San Jose, CA, USA). The stained areas of the nine sections were averaged and expressed as the mean lesion size for each mouse.

To quantitatively evaluate atherosclerotic lesions of the entire aorta from the junction with the heart to the iliac bifurcation, the aorta was removed, cut open, and pinned flat. The aortas were fixed, stained with Oil Red O by floating a styrofoam mount with the aorta facing down in buffered formalin and Oil Red O staining solutions, and examined under a dissecting microscope. Visual estimation of the area covered by Oil Red O staining lesions was performed by two different individuals.

**APPENDIX 4**

**Hepatic and ileal RNA isolation and real-time PCR analysis**

Total RNA was extracted from the liver tissue samples using an RNeasy Mini Kit (Qiagen, Tokyo, Japan). mRNA levels of sterol regulatory element-binding transcription factor 1c (SREBf1c), SREBf2, fatty acid synthase (FAS), acetyl-CoA carboxylase 1 (ACC1), stearoyl-CoA desaturase 1 (SCD1), small heterodimer partner (SHP), bile salt export pump (BSEP), carnitine palmitoyltransferase 1 (CPT1a), cytochrome P450 7A1 (CYP7a1), microsomal triglyceride transfer protein (MTTP), interleukin-6(IL-6), and tumor necrosis factor-α (TNFα) were determined in liver tissue using fluorescence-based reverse transcription-PCR and an ABI PRISM 7700 Sequence Detection System (Life Technologies, Carlsbad, CA, USA). Apical sodium-dependent bile acid transporter (ASBT), FGF15 (fibroblast growth factor 15) expression were also measured in the ileal samples using the same method. All primers were purchased from Sigma-Aldrich (Shinagawa-ku, Japan).

**APPENDIX 5**

**Liquid chromatography-tandem mass spectrometry (LC–MS)**

Analytical standards for the targeted bile acids and deuterated internal standards were purchased from Sigma-Aldrich and Steraloids. Individual stock solutions (100 μM) were prepared in methanol and further diluted with methanol to prepare a calibration standard. A one-point calibration standard (1 μM) was prepared and injected four times. All other reagents were of analytical grade and the solvents used were of LC–MS grade (Wako Chemicals). Human plasma (50 μL)—consisting of a pool of healthy anonymous donors—were mixed with 10 μL of internal standard solution (10 μM) in methanol. Subsequently, 30 μL of aqueous hydrochloric acid solution (1 M) was added, followed by 910 μL of acetonitrile. After vortexing for 1 min, the samples were centrifuged at 14 000 × *g* for 15 min. The supernatant (850 μL) was transferred to a new microtube and evaporated using a vacuum concentrator. Reconstituted extracts with 100 μL methanol were sonicated for 10 min, centrifuged at 14 000 × g for 15 min, and transferred to a vial with a glass insert for analysis using a Nexera X2 UHPLC system coupled with an LCMS-8060 triple quad mass spectrometer (Shimadzu, Kyoto, Japan).

**References**

1. Yan Y, Kolachala V, Dalmasso G, Nguyen H, Laroui H, Sitaraman SV et al. Temporal and spatial analysis of clinical and molecular parameters in dextran sodium sulfate induced colitis. PLOS ONE. 2009;4:e6073. doi:10.1371/journal.pone.0006073.

2. Schneider CA, Rasband WS, Eliceiri KW. NIH Image to ImageJ: 25 years of image analysis. Nat Methods. 2012;9:671–675. doi:10.1038/nmeth.2089.

3. Folch J, Lees M, Sloane Stanley GH. A simple method for the isolation and purification of total lipides from animal tissues. J Biol Chem. 1957;226:497–509.

4. Asai A, Nagao M, Kawahara M, Shuto Y, Sugihara H, Oikawa S. Effect of impaired glucose tolerance on atherosclerotic lesion formation: an evaluation in selectively bred mice with different susceptibilities to glucose intolerance. Atherosclerosis. 2013;231:421–426. doi:10.1016/j.atherosclerosis.2013.10.009.

5. Paigen B, Morrow A, Holmes PA, Mitchell D, Williams RA. Quantitative assessment of atherosclerotic lesions in mice. Atherosclerosis. 1987;68:231–240. doi:10.1016/0021-9150(87)90202-4.

**References**

1 Yan, Y. *et al.* Temporal and spatial analysis of clinical and molecular parameters in dextran sodium sulfate induced colitis. PLoS One 4, e6073, doi:[10.1371/journal.pone.0006073](https://doi.org/10.1371/journal.pone.0006073) (2009).

2 Schneider, C. A., Rasband, W. S. & Eliceiri, K. W. NIH Image to ImageJ: 25 years of image analysis. Nat Methods 9, 671-675, doi:[10.1038/nmeth.2089](https://doi.org/10.1038/nmeth.2089) (2012).

3 Folch, J., Lees, M. & Sloane Stanley, G. H. A simple method for the isolation and purification of total lipides from animal tissues. J. Biol. Chem. 226, 497-509 (1957).

4 Asai, A. *et al.* Effect of impaired glucose tolerance on atherosclerotic lesion formation: an evaluation in selectively bred mice with different susceptibilities to glucose intolerance. Atherosclerosis 231, 421-426, doi:[10.1016/j.atherosclerosis.2013.10.009](https://doi.org/10.1016/j.atherosclerosis.2013.10.009) (2013).

5 Paigen, B., Morrow, A., Holmes, P. A., Mitchell, D. & Williams, R. A. Quantitative assessment of atherosclerotic lesions in mice. Atherosclerosis 68, 231-240, doi:[10.1016/0021-9150(87)90202-4](https://doi.org/10.1016/0021-9150(87)90202-4) (1987).
